# RIG‐I‐Inducing Small Molecules Potently Inhibit HMA‐Resistant AML Through Igniting the Overloaded dsRNA Arsenal

**DOI:** 10.1002/advs.202414477

**Published:** 2025-06-26

**Authors:** Xueqin Chen, Jiaqi Wu, Yuntong Li, Jiayu Huang, Xiangqin Weng, Jiale Wu, Shujun Xiao, Huaxin Song, Zhengyuan Wang, Ni Yan, Fangfang Shi, Derun Zheng, Kai Tan, Hesong Zhang, Jingyi Cui, Wen Wu, Wei Wu, Sujiang Zhang, Min Lu

**Affiliations:** ^1^ Shanghai Institute of Hematology State Key Laboratory of Medical Genomics National Research Center for Translational Medicine at Shanghai Ruijin Hospital Shanghai Jiao Tong University School of Medicine Shanghai 200025 China; ^2^ School of Life Sciences and Biotechnology Shanghai Jiao Tong University Shanghai 200240 China; ^3^ Shanghai Ninth People's Hospital Shanghai JiaoTong University School of Medicine Shanghai 200011 China

**Keywords:** anticancer immune response, acute myeloid leukemia, hypomethylating agent, interferon, RIG‐I

## Abstract

DNA hypomethylating agents (HMAs) are widely used to treat acute myeloid leukemia (AML)/myelodysplastic syndrome (MDS), but most treated patients relapse and lack standard treatment options. Using high‐throughput screening, the approved all‐trans retinoic acid (ATRA) is identified that exhibit high selectivity in killing HMA‐resistant AML cells compared to parental cells. Mechanistically, HMA‐resistant cells are overloaded with DNA hypomethylation‐associated endogenous viral double‐stranded RNA (dsRNA) which, however, fails to trigger an anticancer interferon (IFN) immune response due to downregulation of dsRNA sensor retinoic acid‐inducible gene I (RIG‐I). ATRA compensates for RIG‐I expression, thereby re‐triggering IFN response and potently inhibiting HMA‐resistant AML cell lines, xenograft mice, and patient‐derived primary cells. A library of potential RIG‐I‐inducing compounds is rationally constructed and screened, in which the approved M3 AML treatment drug tamibarotene (TAM) exhibits strikingly 28036‐fold selectivity and 779 pm IC_50_ in killing HMA‐resistant AML cells. ATRA and TAM do not selectively inhibit p53‐mutant cancer cells. Together, this study uncovers a common resistance mechanism in HMA‐treated AML patients and, in addition, provides highly potent and selective agents that can overcome resistance through re‐triggering IFN anticancer immune response.

## Graphical abstract

This study reveals a novel mechanism of resistance to DNA hypomethylating agents (HMAs) in acute myeloid leukemia (AML), and identifies all‐trans retinoic acid (ATRA) and tamibarotene (TAM) as potent agents that overcome HMA resistance by reactivating the interferon anticancer immune response. The findings offer promising therapeutic strategies for HMA‐resistant AML patients.

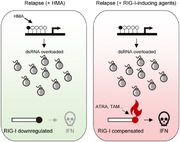

## 1 Introduction

The DNA hypomethylating agents (HMAs) decitabine (DAC) and azacitidine (AZA) are widely used in the treatment of acute myeloid leukemia (AML) and myelodysplastic syndrome (MDS). HMAs were reported to demethylate and upregulate endogenous viral double‐stranded RNA (dsRNA), which was detected by dsRNA sensors, triggering interferon (IFN) anticancer immune responses.^[^ ^1^, ^2^, ^3^ ^]^ DAC has been reported to have striking efficacy in the treatment of some AML and MDS subtypes, such as the highly refractory *TP53*‐mutant AML/MDS.^[^ ^4^ ^]^ Despite the widely recognized efficacy of the HMA, most HMA‐treated AML/MDS patients relapsed.^[^ ^4^, ^5^, ^6^ ^]^

Currently, there are no standard treatment options for HMA‐resistant AML/MDS patients. Next‐generation HMAs such as guadecitabine are being developed, with efficacy observed in two phase II trials.^[^ ^7^, ^8^ ^]^ However, given the same DNA hypomethylation‐dsRNA‐IFN mechanism of action (MoA) shared among HMAs, these new‐generation HMAs may have comprised efficacy in DAC and AZA‐resistant patients. In support of this, switching between HMAs, for example from DAC to AZA or vice versa, does not significantly benefit patients who relapse on initial HMA treatment.^[^ ^9^, ^10^ ^]^ An alternative is to identify HMA resistance mechanism, based on which rationally design a completely new treatment regimen.

The exact molecular mechanisms underlying HMA resistance are not fully understood and several factors have been proposed to be involved, including the inactivation of deoxycytidine kinase (DCK) and uridine cytidine kinase (UCK), the enzymes that convert DAC and AZA into active metabolites in the cells,^[^ ^11^, ^12^ ^]^ and upregulation of cytidine deaminase (CDA), the enzyme responsible for inactivating DAC and AZA in the cells.^[^ ^13^ ^]^ These dysregulated HMA metabolisms effectively abolish DNA demethylating effects of HMAs, which are thought to be associated with patient relapse. However, most of these resistance mechanisms have only been reported in preclinical models and lack clear support in the clinics. For example, normal metabolism of HMA drugs^[^ ^14^, ^15^ ^]^ and DNA hypomethylation^[^ ^14^ ^]^ were still observed in relapsed patients. However, these two upstream events failed to trigger the downstream inflammatory and IFN anticancer immune responses in the relapse patients,^[^ ^16^, ^17^ ^]^ with the mechanism(s) largely unknown.

Here we uncovered that downregulation of retinoic acid‐inducible gene I (*RIG‐I*)^[^ ^18^ ^]^ as an HMA‐resistant mechanism underlying the failure of HMA in triggering IFN anticancer immune responses in AML cell lines, xenograft mice, and patient‐derived primary cells. In addition, we rationally obtained a few agents that can re‐trigger IFN immune response, including an approved M3 AML drug with striking pmol‐level activity and 28036‐fold selectivity in killing HMA‐resistant AML cells. Our study provides promising agents that can be straightforward trialed for HMA‐resistant AML/MDS patients.

## 2 Results

### 2.1 High‐Throughput Screening Identified ATRA as a Potent and Selective Inhibitor of HMA‐Resistant AML Cells

**Figure 1:**
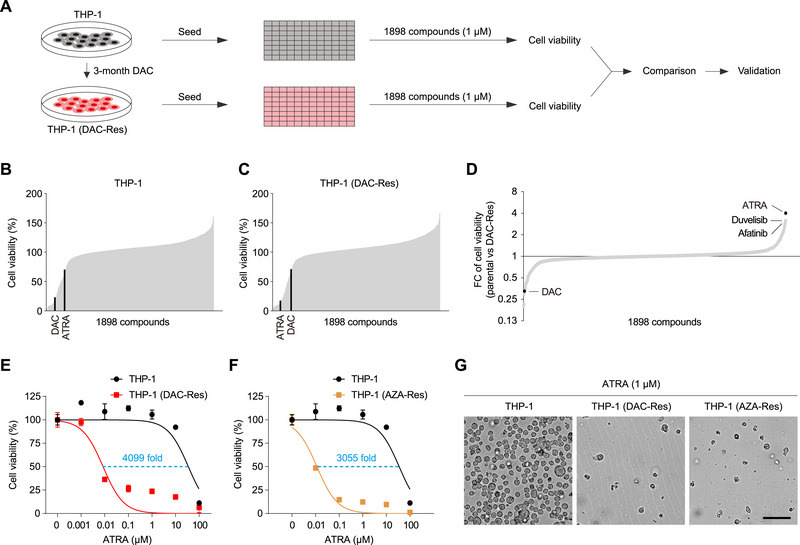
High‐throughput screening identified ATRA as a potent and selective inhibitor of HMA‐resistant AML cells. A) Schematic overview of high‐throughput screening in native and DAC‐resistant THP‐1 cells. Cells were seeded in 384‐well plates and treated with 1898 FDA‐approved drugs at a screening concentration of 1 µm for 5 days. Cell viability was measured for comparison between the two cell lines. B,C) Cell viability of drug‐treated parental (B) and DAC‐resistant (C) THP‐1 cell lines. Drugs are ranked according to cell viability. D) Fold change (FC) of cell viability between the two cell lines for each screened drug. Drugs were ranked according to FC. E,F) The indicated cells were treated with increasing doses of ATRA for 72 h, followed by determination of cell viability. IC_50_ values were calculated using non‐linear curve fitting in GraphPad Prism 8.0. The IC_50_ shift (fold) was calculated. G) Phase‐contrast images of the indicated cells treated with 1 µm ATRA for 72 h. Scale bar, 50 µm. Error bars represent mean ± SD (*n* = 3 biological replicates).

We first screened for 1898 Food and Drug Administration (FDA)‐approved drug (Table S1, Supporting Information) that can selectively kill DAC‐resistant AML cells compared to parental cells (**Figure** **1**A). DAC‐resistant THP‐1 cells were generated by long‐term DAC treatment (Figure 1A). As expected, DAC exhibited much lower cytotoxicity in killing DAC‐resistant THP‐1 cells compared to parental cells (Figure S1A, Supporting Information). High‐throughput screening, based on cell viability after treatment with 1898 FDA‐approved small molecule drugs at 1 µm concentration in parental (Figure 1B) and DAC‐resistant (Figure 1C) THP‐1 cells, identified a few drugs exhibiting selectivity in killing DAC‐resistant cells (Figure 1D, the top right dots). All‐trans retinoic acid (ATRA), duvelisib, and afatinib exhibited the highest selectivity in killing DAC‐resistant cells. As expected, DAC exhibited selectivity in killing parental cells in our screen. We confirmed the selectivity of 1 µm ATRA, duvelisib and afatinib in killing DAC‐resistant THP‐1 cells with three biological replicates (Figure S1B, Supporting Information, *P* < 0.01). Interestingly, 1 µm ATRA, duvelisib, and afatinib also exhibit selectivity in killing AZA‐resistant THP‐1 cells (Figure S1C, Supporting Information, *P* < 0.01), implying that the two resistant THP‐1 cells share similar re‐sensitization mechanisms.

We next characterized the top hit, ATRA, for its killing of HMA‐resistant AML cell lines. In THP‐1 cells, ATRA exhibited high potency (IC_50_ = 8.48 nm) and high selectivity (4099‐fold difference between IC_50_ values) in killing DAC‐resistant THP‐1 cells compared to parental cells (Figure 1E). ATRA also exhibited high potency and selectivity in killing AZA‐resistant THP‐1 cells (Figure 1F, IC_50_ = 11.37 nm, 3055‐fold difference). Treatment with 1 µm ATRA for 72 h resulted in the death of almost all DAC‐ and AZA‐resistant THP‐1 cells, with no apparent effect on parental cells (Figure 1G). ATRA also selectively killed the DAC‐resistant AML cell lines OCI‐AML3 and HL‐60, with 1270‐ and 404‐fold selectivity observed, respectively (Figure S1D,E, Supporting Information). In summary, we have identified ATRA that exhibited high potency and selectivity in killing HMA‐resistant AML cell lines.

### 2.2 Downregulated RIG‐I Fails to Trigger IFN Anticancer Immune Response in HMA‐Resistant AML

**Figure 2:**
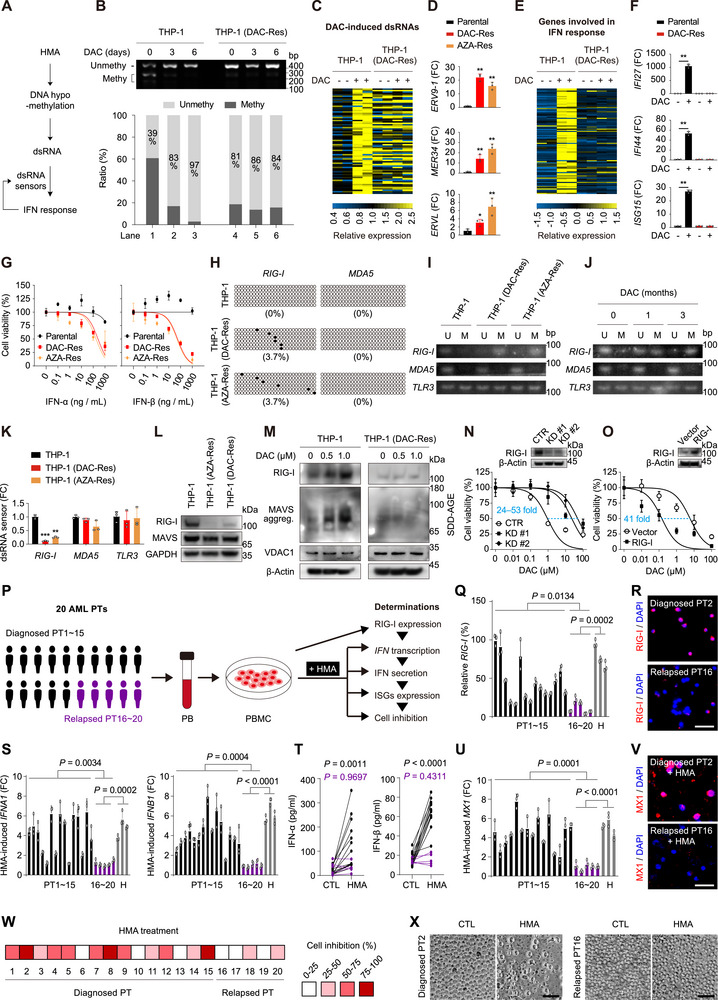
Downregulated RIG‐I fails to trigger IFN anticancer immune response in HMA‐resistant AML. A) Schematic illustration of the IFN immune positive feedback response triggered by HMA through DNA demethylation, dsRNA overloading and detection by dsRNA sensors. B) COBRA‐*LINE‐1* determination of global DNA methylation levels in the indicated cells. The signal density of unmethylated and methylated DNA was quantified using ImageJ in the lower panel. C) The indicated cells were treated the indicated compounds with two replicates, followed by RNA‐seq. Heatmap shows the relative expression levels of DAC‐induced dsRNAs (fold change > 2 and *P* < 0.05 in DAC‐treated parental THP‐1 cells compared to untreated parental cells, *n* = 74) in parental and DAC‐resistant THP‐1 cells. For each dsRNA, expression is normalized to the mean level of the two DAC‐untreated parental THP‐1 samples. D) qRT‐PCR determination of mRNA levels of dsRNAs in the indicated THP‐1 cells. E) Heatmap showing relative expression levels of genes involved in IFN‐α response (hallmark gene sets). RNA‐seq data are derived from (C). F) qRT‐PCR determination of mRNA levels of the indicated ISGs in the indicated THP‐1 cells after 1 µm DAC treatment for 72 h. G) The indicated THP‐1 cells were treated with increasing doses of IFN‐α or IFN‐β for 72 h, followed by determination of cell viability. H) Promoter methylation level detected by bisulfite sequencing in the indicated cells. I,J) Methylation‐specific PCR detecting the indicated promoters in the indicated cells (I) or in THP‐1 cells treated with the IC_90_ dose of DAC for 0–3 months (J). U, unmethylated alleles; M, methylated alleles. K) qRT‐PCR determination of mRNA levels of dsRNA sensors in the indicated cells. L) Immunoblotting of RIG‐I and MAVS protein levels in the indicated cells. M) Immunoblotting of the indicated proteins in the indicated cells treated with DAC for 24 h. Cell mitochondria were isolated for semi‐denaturing detergent agarose gel electrophoresis (SDD‐AGE), and whole‐cell lysates were used for SDS‐PAGE. N,O) Dose‐response curves (bottom) on DAC of RIG‐I‐knockdown (N) or RIG‐I‐overexpressed (O) THP‐1 cells, immunoblotting (top) of RIG‐I is shown. P) Schematic of the experiments designed to investigate RIG‐I expression and sensitivity to HMA in primary PBMCs. PB was collected from 15 diagnosed AML patients and 5 AML patients who relapsed after HMA (DAC or AZA) treatment, followed by PBMC isolation, tissue culture and the designed determinations. The cultured PBMCs from relapsed patients were treated with the HMA (DAC or AZA) corresponding to that used in the patients in the clinics. Q) qRT‐PCR determination of *RIG‐I* in the indicated primary PBMCs. R) RIG‐I immunostaining for the primary PBMCs derived from the representative diagnosed patient #2 and relapsed patient #16. S–X) The cultured PBMCs were treated with 1 µm HMA for 72 h, followed by the designed determinations. S) qRT‐PCR determination of *IFNA1* and *IFNB1*. T) Culture medium was collected and assayed by ELISA for IFN‐α and IFN‐β secretion. U) qRT‐PCR determination of *MX1*. V) MX1 immunostaining of representative primary PBMCs. W) The indicated cells were counted visually under a microscope and the percentage of cell inhibition was color‐coded by quartile. X) Phase‐contrast images of the indicated representative primary PBMCs. PT, patient. H, healthy people. FC, fold change. Scale bar, 50 µm. Error bars represent mean ± SD (*n* = 3 biological replicates, *P < 0.05, **P < 0.01, ***P < 0.001).

HMA demethylates DNA and upregulates endogenous viral dsRNA, which is sensed by dsRNA sensors, triggering IFN anticancer immune response in various solid tumors.^[^ ^1^, ^2^, ^3^ ^]^ Interestingly, the three well reported dsRNA sensors—RIG‐I preferentially recognizing short dsRNA (<1 kbp), melanoma differentiation‐associated gene 5 (MDA5) preferentially recognizing long dsRNA (>2 kbp) in the cytoplasm, and toll‐like receptor 3 (TLR3) preferentially recognizing dsRNA outside the cell membrane or in the endosome,^[^ ^19^, ^20^ ^]^ are also induced by the IFN response,^[^ ^1^, ^2^, ^21^, ^22^, ^23^ ^]^ forming positive feedback (**Figure** **2**A). It is likely that this signaling also exists in AML, however, is blocked somewhere, leading to HMA resistance. We therefore focused on this signaling pathway to investigate the mechanism of HMA resistance. In combined bisulfite restriction analysis (COBRA) of human long interspersed elements‐1 (*LINE‐1*),^[^ ^24^ ^]^ 3–6 days of DAC treatment induced global DNA hypomethylation in parental THP‐1 cells as expected (Figure 2B, lane 1 vs 2, 3). Interestingly, the global DNA methylation level is maintained at a hypomethylation level in DAC‐resistant cells (Figure 2B, lane 1 vs 4), reflecting the deep and long‐term epigenetic memory.^[^ ^25^ ^]^ Furthermore, RNA‐seq results revealed that DNA hypomethylation‐induced dsRNAs were maintained in overloaded state in DAC‐resistant cells (Figure 2C, lanes 5, 6 vs lanes 1, 2), albeit to a relatively lower extent as in DAC‐treated parental cells (lanes 3, 4 vs lanes 1, 2). Quantitatively, 16 dsRNAs were significantly overloaded in DAC‐resistant cells, comparable to the 24 dsRNAs overloaded in DAC‐treated parental cells (Figure S2A, Supporting Information). In gene set enrichment analysis (GSEA), “cellular response to dsRNA” was up‐regulated in DAC‐resistant cells, as the effect of DAC treatment on the parental cells (Figure S2B, Supporting Information, both *P* = 0.000). We confirmed by qRT‐PCR that the representative dsRNAs *ERV9‐1*, *MER34*, and *ERVL* were highly over‐expressed in both DAC‐ and AZA‐resistant THP‐1 cells (Figure 2D). Despite of the overloaded state, the dsRNAs failed to upregulate genes involved in the IFN response in RNA‐seq (Figure 2E, lanes 5, 6 vs lanes 1, 2). Of the 58 IFN‐stimulated genes (ISGs) that were significantly upregulated by DAC in parental cells, none were significantly upregulated in DAC‐resistant cells (Figure S2C, Supporting Information). In DAC‐resistant cells, genes involved in “IFN‐α response” were not significantly upregulated upon DAC treatment, in striking contrast to the strong upregulation upon DAC treatment of parental cells (Figure S2D, Supporting Information, *P* values: 0.825 vs 0.000). We confirmed by qRT‐PCR that the representative ISGs *IFI27*, *IFI44*, and *ISG15* could not be upregulated by DAC in the DAC‐resistant cells (Figure 2F). Compared with parental cells, both IFN‐α and IFN‐β selectively killed DAC‐ and AZA‐resistant cells (Figure 2G), supporting that the HMA‐resistant cells are overloaded with dsRNA that can be spark by the added IFN‐α/β.

The failure of the overloaded dsRNA in triggering IFN immune response may be caused by dysfunctional dsRNA sensors (Figure 2A). Among the three dsRNA sensors, *RIG‐I*, but not *MDA5* or *TLR3*, the promoter methylation levels of which were substantially increased in DAC‐ and AZA‐resistant THP‐1 cells (Figure 2H,I). Additionally, similar findings were observed in OCI‐AML3 and HL‐60 cells (Figure S2E,F, Supporting Information). Notably, the *RIG‐I* promoter has been gradually hypermethylated during 3 months under DAC pressure, indicating a process of stepwise selection of cells with *RIG‐I* promoter hypermethylation (Figure 2J). Moreover, *RIG‐I* was dramatically downregulated at the mRNA level in DAC‐resistant THP‐1 cells (Figure 2K). *RIG‐I* downregulation was also observed in DAC‐resistant OCI‐AML3 and HL‐60 cells (Figure S2G, Supporting Information). We confirmed the downregulation of RIG‐I protein in both DAC‐resistant and AZA‐resistant THP‐1 cells by immunoblotting (Figure 2L) and immunostaining (Figure S2H, Supporting Information). RIG‐I was also downregulated in DAC‐resistant OCI‐AML3 and HL‐60 cells (Figure S2H,I, Supporting Information). MAVS aggregation is a marker of RIG‐I activation upon dsRNA recognition.^[^ ^26^ ^]^ Indeed, DAC effectively induced MAVS aggregation in parental THP‐1 cells, however, not in the RIG‐I‐downregulated DAC‐resistant cells (Figure 2M). We next modulated RIG‐I levels and determined sensitivity of THP‐1 cells to DAC treatment. As expected, RIG‐I‐knockdown cells, which mimic DAC‐resistant cells, is resistant to DAC treatment (Figure 2N, IC_50_ increases by 24–53 folds). In contrast, RIG‐I overexpression sensitized THP‐1 cells to DAC treatment (Figure 2O, IC_50_ decreases by 41 folds). Taken together, RIG‐I downregulation leads to the failure of overloaded dsRNAs in triggering IFN immune response in HMA‐resistant AML cells.

We next asked whether the identified HMA resistance mechanism existed in AML patients who relapsed after HMA treatment. We followed up 20 AML patients at diagnosis (n = 15) or after relapse from HMA treatment (n = 5, 3 from DAC treatment and 2 from AZA treatment) (Figure S2J, Supporting Information). Peripheral blood (PB) was collected from these patients and primary peripheral blood mononuclear cells (PBMCs) were prepared and cultured, followed by RIG‐I determination (Figure 2P). The cultured PBMCs were further treated with HMAs (DAC or AZA) corresponding to the one used in the patients (Figure 2P). Compared to PBMCs from diagnosed patients or healthy people, those from relapsed patients had lower *RIG‐I* mRNA levels by qRT‐PCR (Figure 2Q). In immunofluorescence staining, PBMCs from relapsed patients expressed much lower RIG‐I, as compared to that from diagnosed patients (Figure 2R; Figure S2K, Supporting Information; samples from the representative patients #2 and #16 are shown). Notably, HMA treatment potently promoted the transcription (Figure 2S) and secretion (Figure 2T) of IFN‐α and IFN‐β in the cultured PBMCs derived from the 15 diagnosed patients, but not from the 5 relapsed patients. As expected, HMA treatment potently promoted transcription of the ISGs *MX1*, *IFI27*, and *ISG15* in qRT‐PCR (Figure 2U; Figure S2L, Supporting Information), as well as the expression of MX1 protein in immunofluorescence staining (Figure 2V; Figure S2M, Supporting Information; representative samples shown) in the PBMCs derived from diagnosed patients rather than relapsed patients. In the cytotoxicity assay, 1 µm HMA inhibited more than 50% of the cell viability in 9 of the 15 PBMC samples derived from diagnosed patients, but only 0 of the 5 PBMC samples derived from relapsed patients (Figure 2W). Upon treatment with 1 µm HMA, PBMCs from the representative diagnosed patient #2 died severely, whereas the cells from the relapsed patient #16 was not obviously affected (Figure 2X). Together, RIG‐I downregulation mediated silencing of the IFN immune response in HMA‐relapse AML patients.

### 2.3 ATRA Compensates for RIG‐I Expression and Re‐triggers IFN Anticancer Immune Response in HMA‐resistant AML

**Figure 3:**
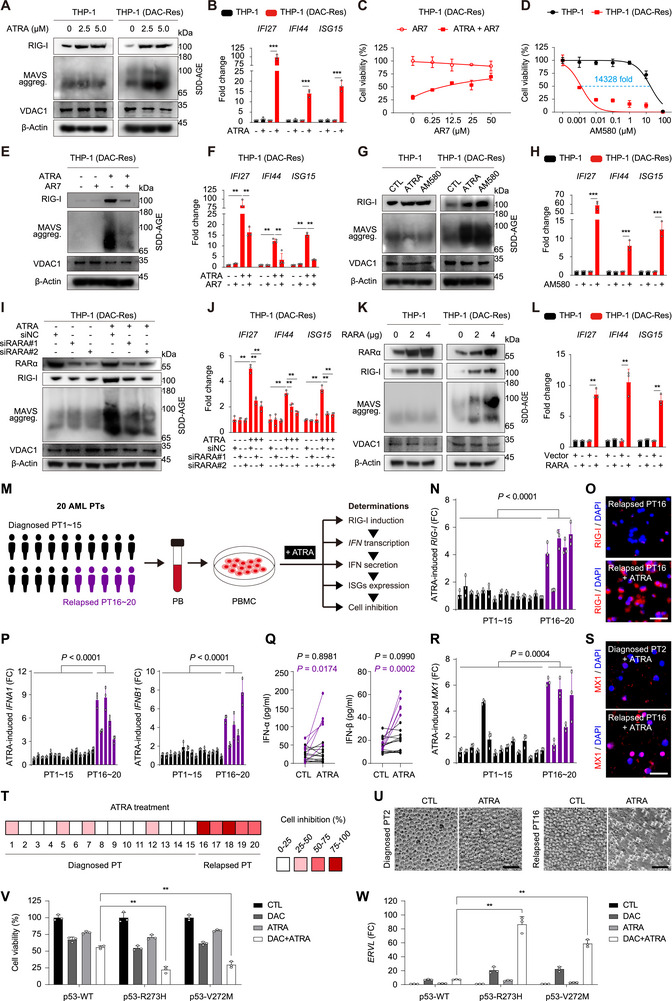
ATRA compensates for RIG‐I expression and re‐triggers IFN anticancer immune response in HMA‐resistant AML. A) Immunoblotting of the indicated proteins in the indicated cells treated with ATRA for 24 h by SDD‐AGE and SDS‐PAGE. B) qRT‐PCR determination of the mRNA levels of the indicated ISGs in the indicated cells after treatment with 1 µm ATRA for 72 h. C) The indicated cells were pretreated with increasing doses of AR7 for 4 h, and then treated with or without 1 µm ATRA for 72 h, followed by determination of cell viability. D) The indicated cells were treated with increasing doses of AM580 for 72 h, followed by determination of cell viability. E,F) The indicated cells were pretreated with 10 µm AR7 for 4 h, followed by treatment with 1 µM ATRA for 24 h and immunoblotting (E) or treatment with 1 µm ATRA for 72 h and qRT‐PCR determination (F). G,H) The indicated cells were cultured, followed by treatment with 1 µm ATRA or AM580 for 24 h and immunoblotting (G) or treatment with 1 µm AM580 for 72 h and qRT‐PCR determination (H). I,J) The indicated cells were transfected with siRNA targeting RARα for 24 h and then treated with 1 µm ATRA for 48 h, followed by immunoblotting (I) and qRT‐PCR determination (J). K,L) Cells were transfected with empty vector or plasmid encoding RARα for 48 h, followed by immunoblotting (K) and qRT‐PCR determination (L). M) Schematic of the experiments designed to investigate the sensitivity of primary PBMCs to ATRA. N–U) The cultured PBMCs were treated with 1 µm ATRA for 72 h, followed by the designed experiments. N) qRT‐PCR determination of *RIG‐I*. O) RIG‐I immunostaining of two representative samples. P) qRT‐PCR determination of *IFNA1* and *IFNB1*. Q) Supernatants were collected and assayed by ELISA for IFN‐α and IFN‐β production. R) qRT‐PCR determination of *MX1*. S) MX1 immunostaining of two representative samples. T) The indicated cells were counted visually under a microscope and the percentage of cell inhibition was colour‐coded by quartile. U) Phase‐contrast images of the indicated primary PBMCs. V,W) The indicated SJSA‐1 cells with different p53 status, generated via CRISPR genome‐editing technology, were treated with 1 µm DAC and 10 µm ATRA for 72 h, followed by determination of cell viability (V) and qRT‐PCR determination (W). PT, patient. FC, fold change. Scale bar, 50 µm. Error bars represent mean ± SD (*n* = 3 biological replicates, **P < 0.01, ***P < 0.001).

Coincidently, *RIG‐I* is named because it is highly induced by ATRA^[^ ^18^ ^]^—the most selective FDA‐approved drug in killing HMA‐resistant cells in our screen (Figure 1D). It is therefore tempting to hypothesize that ATRA kills HMA‐resistant AML by compensating for RIG‐I expression. Supporting this hypothesis, level of the downregulated RIG‐I in HMA‐resistant THP‐1 cells can be compensated for by ATRA treatment to normal level, accompanying with re‐induced MAVS aggregation (**Figure** **3**A). Consequently, the representative ISGs, including *IFI27*, *IFI44*, and *ISG15*, could be reactivated by ATRA treatment in DAC‐ and AZA‐resistant cells (Figure 3B; Figure S3A, Supporting Information, respectively; all *P* < 0.001).

ATRA binds its receptor retinoic acid receptor alpha (RARα),^[^ ^27^ ^]^ leading to RIG‐I upregulation.^[^ ^18^ ^]^ This allows us to modulate RIG‐I levels using the reported RARα inhibitor AR7^[^ ^28^ ^]^ and agonist AM580,^[^ ^29^ ^]^ to block or mimic ATRA‐induced death of HMA‐resistant cells, respectively. As expected, AR7 effectively inhibited ATRA‐induced death of HMA‐resistant cells in a dose‐dependent manner (Figure 3C; Figure S3B, Supporting Information), whereas AM580 mimicked the ability of ATRA to selectively kill DAC‐resistant cells (Figure 3D, 14328‐fold change). Mechanistically, AR7 blocked ATRA‐induced RIG‐I upregulation and MAVS aggregation (Figure 3E) and subsequently impaired activation of representative ISGs (Figure 3F; Figure S3C, Supporting Information) in ATRA‐treated HMA‐resistant cells. In contrast, AM580 compensated for RIG‐I expression and MAVS aggregation (Figure 3G) and upregulated ISGs (Figure 3H) in DAC‐resistant cells. To ensure that the above observations were not caused by off‐target effects of AR7 and AM580, we knocked down or overexpressed RARα to block or promote RIG‐I expression, respectively. Consistent with the results observed with AR7 treatment, RARα knockdown effectively blocked RIG‐I expression and MAVS aggregation (Figure 3I) and subsequently impaired activation of representative ISGs (Figure 3J) in ATRA‐treated DAC‐resistant cells. In contrast, RARα overexpression effectively induced RIG‐I expression and MAVS aggregation (Figure 3K) and upregulated ISGs (Figure 3L) in DAC‐resistant cells. Similar results of ISGs expression were observed in AZA‐resistant THP‐1 cells (Figure S3D,E, Supporting Information).

In parallel experiments using the cultured primary PBMCs derived from the abovementioned 20 AML patients, we treated the cells with ATRA, followed by determination of RIG‐I expression, IFN signaling, and cell inhibition (Figure 3M). ATRA‐induced *RIG‐I* transcription in qRT‐PCR (Figure 3N) and protein expression in immunofluorescence staining (representative image seen in Figure 3O; Figure S3F, Supporting Information) were significantly higher in PBMCs from relapsed patients than in those from diagnosed patients. Meanwhile, ATRA potently promoted the transcriptions (Figure 3P) and secretions (Figure 3Q) of IFN‐α and IFN‐β in PBMCs from relapsed patients, but not in those from diagnosed patients. Similar results were observed when we detected the transcription of the ISGs *MX1*, *IFI27*, and *ISG15* (Figure 3R; Figure S3G, Supporting Information), as well as the expression of MX1 protein (Figure 3S; Figure S3H, Supporting Information). In the cytotoxicity assay, 1 µM ATRA treatment for 72 h kills more than 50% of cells in all 5 PBMC samples derived from relapsed patients and in 0 of 15 samples from diagnosed patients (Figure 3T; representative cell morphology image seen in Figure 3U). Taken together, ATRA selectively kills HMA‐resistant AML cells by compensating for RIG‐I expression, in both cultured cell lines and patient‐derived primary cells.

Of note, recent work has reported the cooperative induction of endogenous retroviruses by DAC in combination with ATRA, in AML cell lines of different *TP53* status,^[^ ^30^ ^]^ lending further support to the implementation of ATRA in the first‐line treatment for p53‐mutant patients.^[^ ^31^ ^]^ Consistently, we found that the combination of DAC and ATRA caused more cell death (Figure 3V) and induced more endogenous retroviruses dsRNA such as *ERVL* (Figure 3W) in cells with mutant p53 than wild‐type p53.

### 2.4 ATRA Prolongs Survival of HMA‐Resistant AML Xenograft Mice by Re‐Triggering IFN Anticancer Immune Response

**Figure 4:**
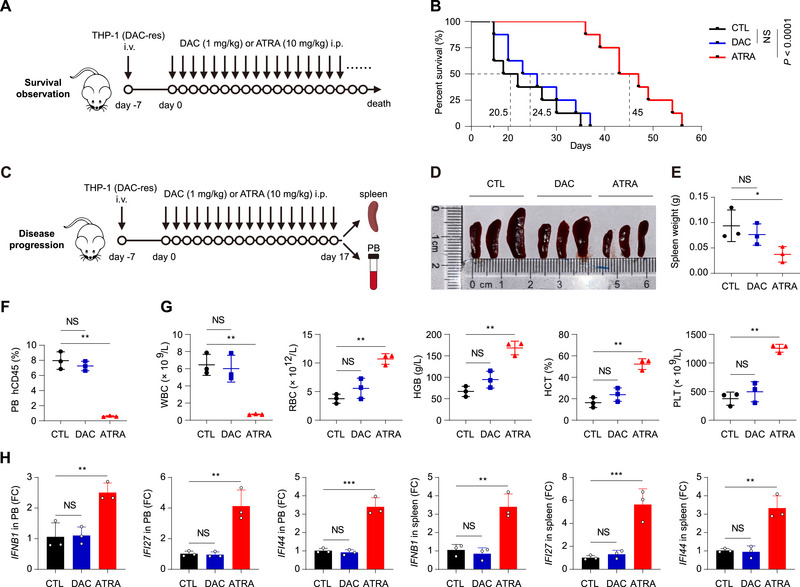
ATRA prolongs survival of HMA‐resistant AML xenograft mice by re‐triggering IFN anticancer immune response. A,B) Schematic of the xenograft study on survival observation. A) NCG mice were inoculated with DAC‐resistant THP‐1 cells via the tail vein (i.v.) on day ‐7. Mice were treated with vehicle, DAC or ATRA intraperitoneally (i.p.) daily from day 0 until death. There were 8 mice in each group. B) Kaplan‐Meier survival curves for the mice in (A). The Log‐rank (Mantel‐Cox) test was used to compare survival between groups in GraphPad Prism 8.0. C–H) Schematic of the xenograft study on disease progression. C) Mice were treated as in (A), except that mice were sacrificed on day 17 and the spleens and PB were harvested for the designed experiment. D) Photograph of the spleens harvested on day 17. E) The weight of the harvested spleens. F) Flow cytometric analysis of the percentage of human CD45‐positive cells (the transplanted DAC‐resistant THP‐1 cells) in the harvested PB. G) Blood counts in the harvested PB. WBC, white blood cells. RBC, red blood cells. HGB, hemoglobin. HCT, hematocrit. PLT, platelets. H) qRT‐PCR determination of the indicated genes in the harvested PB and spleens. FC, fold change. NS, not significant. Error bars represent mean ± SD (*n* = 3 biological replicates, *P < 0.05, **P < 0.01, ***P < 0.001).

We next investigated therapeutic potential of ATRA in the treatment of HMA‐resistant AML cells in vivo. In the survival monitor xenograft model, mice were xenografted with HMA‐resistant THP‐1 cells, followed by DAC or ATRA treatment until death (**Figure** **4**A, *n* = 8 in each group). DAC treatment prolonged median survival of mice from 20.5 to 24.5 days without statistical significance, whereas ATRA treatment dramatically prolonged median survival from 20.5 to 45 days with a high significance (Figure 4B, *P* < 0.0001).

To confirm that the observed survival benefit was associated with attenuated leukemia expansion and re‐triggered IFN signaling, we repeated the survival observation experiment whereas the mice were sacrificed on day 17, followed by spleen and PB harvest and determinations (Figure 4C). ATRA treatment, but not DAC treatment, delayed swelling (Figure 4D) and weight increasing (Figure 4E) of spleen on day 17. In the harvested PB, ATRA treatment, but not DAC treatment, significantly decreased the population of the xenografted human CD45‐positive THP‐1 cells (Figure 4F, *P* < 0.01). Many of the key indices in complete blood count were significantly improved upon ATRA treatment, including suppressed white blood cells (WBC), increased red blood cells (RBC), hemoglobin (HGB), hematocrit (HCT), and platelets (PLT) (Figure 4G, all *P* < 0.01; more indices seen in Figure S4A–C, Supporting Information). In both PB and spleens, the mRNA levels of the representative IFN‐related genes *IFNB1*, *IFI27*, and *IFI44* were all significantly upregulated in the ATRA group (Figure 4H, all *P* < 0.01). Taken together, ATRA has a promising therapeutic effect in the treatment of HMA‐resistant AML xenograft through re‐triggering IFN response, recapitulating the findings in cell lines and patient‐derived primary cells.

### 2.5 Rational Identification of Small Molecules that Kill HMA‐resistant AML Cells with Super Potency and Selectivity

**Figure 5:**
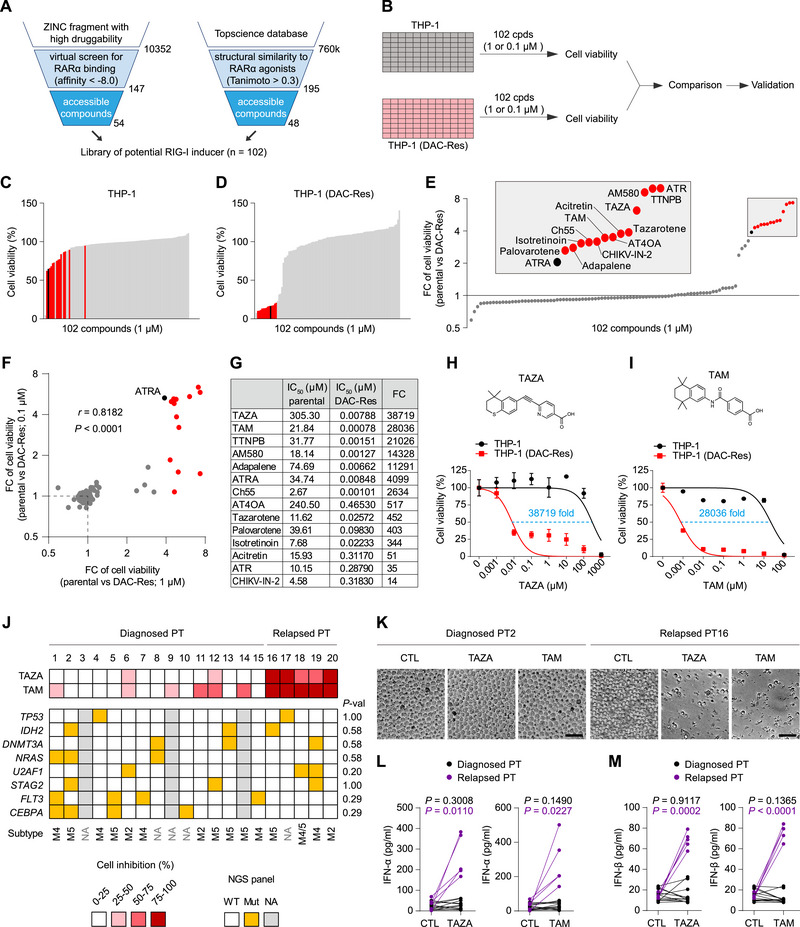
Rational identification of small molecules that kill HMA‐resistant AML cells with super potency and selectivity. A) Workflow of library construction of potential RIG‐I‐inducing small molecules. The 54 commercially available small molecules predicted to bind RARα with high affinity (< −8.0 kcal mol^−1^) in 1‐click Docking and the 48 commercially available small molecules with high structural similarity to the known type I RARα agonists TAM, AM580, tazarotene and adapalene or type II RARα agonists peretinoin, isotretinoin, ATRA and acitretin (Tanimoto score > 0.3) were collected to form the library. B) Schematic overview of compound screening in parental and DAC‐resistant AML cells. Cells were seeded in 384‐well plates and treated with the small molecules from the constructed library in (A) at a screening concentration of 1 or 0.1 µm for 5 days. Cell viability was measured for comparison between the two cell lines. Cpds, compounds. C–E) Results of the screening performed with 1µm compound. C,D) Cell viability of compound‐treated parental (C) and DAC‐resistant (D) THP‐1 cell lines. Compounds are ranked by cell viability. E) FC of cell viability between the two cell lines for each screened drug. Drugs were ranked according to FC. The compounds with higher selectivity than ATRA between the two cell lines are shown as red bars or dots. F) Dot plot comparing FC of cell viability in parental versus DAC‐resistant THP‐1 cells. The viability data were derived from the screening studies using 1 and 0.1 µm compounds. *r* is Pearson's correlation coefficient. G) Summarized IC_50_ values and IC_50_ shift (fold) treated with the indicated compounds in the two indicated cell lines. H,I) Chemical structures of TAZA (H) and TAM (I) and their dose response in the two indicated cell lines. The IC_50_ shift (fold) was calculated. J–M) PBMCs from 20 diagnosed and relapsed AML patients were treated with 1 µm TAZA or TAM for 72 h, followed by the designed experiments. J) The indicated cells were counted visually under a microscope and the percentage of cell inhibition was color‐coded by quartile (top). Genetic mutations and the subtypes of AML were depicted (bottom). For calculating the correlation between the response rate to TAM and the indicated genetic mutations, Fisher's exact test was used and the corresponding *P*‐values were shown in the right panel. NGS, next‐generation sequencing. WT, wild‐type. Mut, mutant. NA, not available. *P*‐val, *P*‐value. K) Phase‐contrast images of representative primary PBMCs. Scale bar, 50 µm. L,M) Culture medium was collected and assayed by ELISA for IFN‐α (L) and IFN‐β (M) production. PT, patient. FC, fold change. Error bars represent mean ± SD (*n* = 3 biological replicates).

Elucidation of HMA resistance mechanism enables us to rationally identify small molecules that may be more potent and selective than ATRA in killing HMA‐resistant AML cells—through constructing and screening library composed of potential RARα agonists. 54 commercially available small molecules predicted to bind RARα with high affinity (< −8.0 kcal mol^−1^) in 1‐click Docking were collected (**Figure** **5**A, left panel; Table S2, Supporting Information). 48 commercially available small molecules with high structural similarity (Tanimoto score > 0.3) to the known type I RARα agonists tamibarotene (TAM), AM580, tazarotene, and adapalene, or the type II RARα agonists peretinoin, isotretinoin, ATRA and acitretin were also collected (Figure 5A, right panel, Figure S5A and Table S2, Supporting Information). The 102 compounds made up a library of potential RIG‐I inducers, which was subsequently screened for their selectivity in killing DAC‐resistant THP‐1 cells compared to parental cells as in Figure 1A (Figure 5B; Table S3, Supporting Information). In the rationally constructed library, up to 13 compounds moderately killed parental THP‐1 cells (Figure 5C, the red lines), but potently killed DAC‐resistant THP‐1 cells (Figure 5D, the red lines) at 1 µm. These 13 compounds exhibit even higher selectivity than ATRA in killing DAC‐resistant cells (Figure 5E, the red dots vs the black dot). We repeated the screening using 0.1 µm of the potential RIG‐I‐inducing compounds (Figure S5B–D, Supporting Information) and identified a generally similar set of compounds (Figure 5F, *r* = 0.8182, *P* < 0.0001).

We then characterized the 13 hits, together with ATRA, in killing DAC‐resistant cells. These 14 compounds exhibited IC_50_ values ranging from 2.67 to 305.30 µm in killing THP‐1 cells and, strikingly, from 0.00078 to 0.46530 µm in killing DAC‐resistant cells (Figure S5E, Supporting Information, summarized in Figure 5G). Among them, tazarotenic acid (TAZA),^[^ ^32^ ^]^ the metabolite of parapsoriasis‐treating tazarotene, exhibited the highest selectivity in killing DAC‐resistant cells (Figure 5H, fold change = 38719), while the M3 AML treatment drug TAM^[^ ^29^ ^]^ exhibited the highest potency in killing DAC‐resistant cells (Figure 5I, IC_50_ = 779 pm).

In primary PBMCs derived from the abovementioned 20 diagnosed and relapsed AML patients, despite genetic mutations and the subtypes of AML varying (Figure 5J, bottom), both TAZA and TAM exhibited high potency and selectivity in killing PBMCs from relapsed patients (Figure 5J). As consistent with the lack of significant correlation (*P* = 1.00) between the response rate to TAM and p53 mutation (Figure 5J, right panel), no difference was observed for the IC_50_ values of TAM between p53‐wild‐type and p53‐mutant cells sharing the same genetic background (Figure S5F, Supporting Information). Treatment with 1 µm TAM for 72 h killed over 75% of PBMCs in all 5 samples from relapsed patients (representative cell morphology shown in Figure 5K). We confirmed that the super potency and selectivity of TAZA and TAM were associated with activation of IFN signaling, as supported by the high‐level secretions of IFN‐α (Figure 5L, 3–104‐fold promotions) and IFN‐β (Figure 5M, 4–7‐fold promotions) in the culture medium of PBMCs harvested from relapsed rather than diagnosed AML patients.

## 3 Discussion

**Figure 6:**
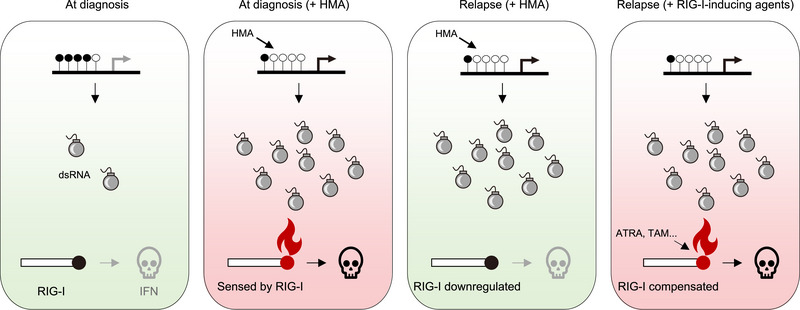
Proposed mechanism underlying how RIG‐I‐inducing agents potently kill HMA‐resistant AML. In diagnosed AML patients, cancer cells remain in a state of DNA hypermethylation and low‐level dsRNA (the first panel). HMA induces DNA hypomethylation, leading to dsRNA overload (bomb icon), which is sensed by RIG‐I (match icon) and triggers IFN anticancer immune response (the second panel). In patients relapsed after HMA treatment, the dsRNA arsenal fails to trigger IFN immune response due to downregulation of RIG‐I (the third panel). Treatment with RIG‐I‐inducing agents, such as ATRA and TAM, compensates for expression of RIG‐I and re‐triggers IFN immune response (the last panel).

HMAs were reported to induce DNA hypomethylation and upregulate endogenous viral dsRNA, which was sensed by dsRNA sensors, triggering IFN anticancer immune response in various solid tumors.^[^ ^1^, ^2^, ^3^ ^]^ This DNA hypomethylation‐dsRNA overloading‐IFN signaling is recapitulated in HMA‐treated AML in the current study (**Figure** **6**, first two panels). It is surprising that HMA‐resistant AML cells are maintained in a state of global DNA hypomethylation and dsRNA overloading, however, it fails to trigger IFN anticancer immune response due to silence of dsRNA sensor RIG‐I (Figure 6, third panel). The next‐generation HMAs share the similar DNA demethylation MoA and thus they may be ineffective in treating DAC and AZA relapse patients due to RIG‐I silence. Here, we identified the RIG‐I‐inducing agents, which employ a distinct MoA—igniting the pre‐existing dsRNA arsenal through compensating for RIG‐I expression—to potently kill HMA‐resistant cells (Figure 6, last panel). It is notable that the revealed resistance mechanism and the achieved re‐sensitization are recapitulated in the AML patient‐derived primary cells, suggesting clinical relevance of our findings.

By special coincidence, out of the 1898 unbiasedly screened FDA‐approved drugs used to treat numerous human diseases, the top 1 hit (ATRA) in selectively killing HMA‐resistant AML is also a drug approved for AML treatment. The rationale underlying such a coincidence is unknown. Since the common action of HMAs and ATRA is to trigger IFN anticancer immune response, albeit one at the upstream DNA demethylation step^[^ ^1^, ^2^, ^3^ ^]^ and the other at the intermediate dsRNA sensing step,^[^ ^33^, ^34^, ^35^ ^]^ a possible explanation for such a coincidence is that AML is a cancer type that is particularly susceptible to IFN immune response. With this speculation, it is possible that modulation at the downstream IFN effect steps, for example direct supplementation of interferon proteins, may also be effective in treating HMA‐resistant AML.

Given the long testing times and high costs of developing new HMA drugs, repurposing TAZA, a metabolite of the approved parapsoriasis drug tazarotene, and the M3 AML treatment drugs ATRA and TAM, represents a rapid and economical drug development strategy. However, drug repurposing has limited success precedents in the clinic—few repurposed drugs are officially included in the published cancer clinical practice guidelines.^[^ ^36^, ^37^ ^]^ The main barriers lie on the lack of therapeutic window, which are caused by the unsatisfactory pharmacokinetics (PK) and pharmacodynamics (PD), when repurposing an old drug from the existing indication to a new indication. Regarding the barrier of PK, the old drug may be insufficient to reach the desired site in the human body when applied in the new indication. Coincidentally, the identified ATRA and TAM have been approved for the treatment of M3 AML. Cancer cells in M3 AML (existing indication) and HMA‐resistant AML/MDS (new indication) both reside in the hematological system, thus removing the barrier of PK during their repurposing. Regarding the barrier of PD, the safe dosage in the human body, although high enough to modulate the established function in the existing indication (herein inducing M3 AML cell differentiation^[^ ^38^, ^39^ ^]^), maybe insufficient to modulate the desired function in the new indication (herein upregulating RIG‐I and triggering IFN immune response). Notably, ATRA, identified in our blind screening, exhibits 8.48 nm IC_50_ and 4099‐fold selectivity in killing HMA‐resistant AML cells. TAM, identified in our rationally designed screening, exhibit striking pM‐level anticancer effect and 28036‐fold selectivity against resistant cells. Such low effective concentrations and high selectivities suggest that their safe dosages in the human body are likely sufficient to trigger IFN immune response and kill HMA‐resistant AML cells in the new indication. With these inherent advantages, ATRA and TAM very likely have sufficient therapeutic window when repurposed for the treatment of HMA‐resistant AML/MDS.

It is notable that several groups have implemented ATRA already as first‐line treatment of AML/MDS patients, combining it with a hypomethylating agent. In Wass's study, the combination of ATRA and tranylcypromine resulted in a 3.3‐month overall survival (OS) and a 20% overall response rate in relapsed/refractory (r/r) AML patients.^[^ ^40^ ^]^ In Cao's study, the combination of ATRA and DAC resulted in a 12.1‐month OS and a 69.4% objective response rate (ORR) in elderly AML patients.^[^ ^41^ ^]^ In Lübbert's study, the combination of ATRA and DAC resulted in an 8.4‐month OS and a 26.1% ORR in elderly AML patients.^[^ ^42^ ^]^ In Gu's study, triplet combination of azacitidine, venetoclax, and ATRA in first‐line AML patients resulted in a 573‐day OS, a 390‐day event free survival (EFS), and a 91% ORR.^[^ ^31^ ^]^ All these exciting outcomes suggest the promising potential of adding ATRA much earlier in the treatment sequence, in order to delay time to secondary resistance, rather than adding it late during treatment, in a clinical trial. On the other hand, we notice that tamibarotene, in combination with azacitidine, did not result in an increase in response rates in two randomized trials (NCT04905407 and NCT04797780). Hence for the time being, ATRA may well be the first candidate for HMA combination trials.

p53 mutation predicts poor prognosis in AML/MDS.^[^ ^43^, ^44^, ^45^ ^]^ In 2013, Leonova et al. reported that HMA‐induced endogenous retroviruses could selectively kill p53‐mutant MEF cells.^[^ ^3^ ^]^ Similar p53 mutant‐preferential antileukemic activity of decitabine, in combination with ATRA in AML upon PML/RARA‐negative AML blasts, was reported in vitro and in vivo.^[^ ^30^, ^46^ ^]^ The effect of HMA in selectively benefiting p53‐mutant AML/MDS patients are also reported in the clinics. In a retrospective study, DAC achieved a 71.4% complete response (CR) rate, and improved OS of p53‐mutant MDS patients to the level of p53‐wild‐type patients.^[^ ^47^ ^]^ Furthermore, Welch et al. reported that AML/MDS patients with *TP53* mutations exhibited higher response rates to DAC, compared to those with wild‐type *TP53* (100% vs 41%) in a prospective clinical trial (NCT01687400).^[^ ^4^ ^]^ It is reported that combined p53 inactivation and HMA‐induced DNA demethylation lead to unsilencing and massive transcription of repeat elements to form dsRNA, followed by triggering of a suicidal type I IFN response.^[^ ^3^, ^48^ ^]^ However, we also note that there is report that HMA does not have selective efficacy in treating p53‐mutant AML/MDS patients.^[^ ^49^ ^]^ Together, as an agent that can boost endogenous retroviruses‐induced IFN antitumor immunity through sensing endogenous retroviruses, ATRA may have potential in combining with HMA to selectively kill p53‐mutant AML/MDS cells, underling the necessity of large‐scale clinical trials for the HMA (with or without ATRA combination) regimen in p53‐mutant AML/MDS patients.

There are no standard treatment options for AML/MDS patients relapsed after HMA treatment. Our study provides an opportunity to rapidly repurpose the approved M3 AML drugs (ATRA and TAM) for treatment of the relapse patients. This repurposing regimen is widely applicable yet needs to be precisely applied—extending to non‐M3 AML but restricting to HMA‐relapse subcohort.

## 4 Experimental Section

### Ethics Statement

All animal experiments described in this study were approved by the Institutional Animal Care and Use Committee (IACUC) of Shanghai Jiao Tong University (approval No. A2019‐004). Peripheral blood was collected with the patients’ written informed consent and approved by the Ethics Committee and Institutional Review Board of Ruijin Hospital.

### Cell Lines

THP‐1, OCI‐AML3, and HL‐60 cells were cultured in RPMI 1640 medium (22400105, Thermo Fisher), HEK293T and SJSA‐1 cells were cultured in DMEM medium (11995073, Thermo Fisher). Both medium are supplemented with 10% fetal bovine serum (FBS), 100 U mL^−1^ penicillin, and 100 mg mL^−1^ streptomycin (15140‐122, Thermo Fisher). OCI‐AML3 cells were purchased from DSMZ, and the other cell lines were purchased from ATCC. All cell lines have been authenticated by the short tandem repeat (STR) assay and were confirmed to be without mycoplasma contamination. The SJSA‐1 cells with different p53 status were generated via CRISPR genome‐editing technology. Peripheral blood mononuclear cells (PBMCs) from AML patients or healthy people were cultured in RPMI 1640 medium, supplemented with 20% FBS, 100 U mL^−1^ penicillin, and 100 mg mL^−1^ streptomycin. Cell cultures were maintained in a 5% CO_2_ humidified incubator at 37 °C.

To generate the HMA‐resistant AML cell lines, the parental cells were exposed to pulsed drug treatment as previously reported.^[^ ^50^ ^]^ Briefly, cells were treated with the IC_90_ dose of HMA until 90% cell death was reached, then cultured in HMA‐free medium until confluence, followed by another round of HMA treatment. The treatment cycles were repeated for 3 months, until significant HMA resistance was observed. Since AZA and DAC are widely considered to be unstable,^[^ ^51^, ^52^ ^]^ the medium containing HMAs was refreshed daily. Every two weeks, the survived cells were treated with increasing doses of HMA for 72 h, followed by determination of cell viability. The IC_50_ and IC_90_ values were then calculated to assess HMA‐resistance level of the cells and guide the subsequent dose adjustment. The cells gradually acquired resistance to HMA through a series of stepwise selections. These selected cells were cultured in HMA‐free medium for at least 2 weeks prior to the experiment.

### Compound Screen

THP‐1 parental and DAC‐resistant cells were screened with an FDA‐approved drug library (n = 1898, 1 µm, Topscience, Table S1, Supporting Information) or potential RIG‐I inducer library (n = 102, 1 µm or 0.1 µm, Table S2, Supporting Information) for 5 days. The negative control, DMSO (D2650, Sigma) was included in each plate. Subsequently, the cell viability was assessed using CellTiter‐Glo (G7573, Promega) according to the manufacturer's instructions.

### Cell Viability Assay

Cell viability was determined using a Cell Counting Kit‐8 (C0005, TargetMol) according to the manufacturer's instructions.

### COBRA

Genomic DNA was extracted cells using a Blood Genomic DNA Mini Kit (CW2087M, Cwbiotech). COBRA of *LINE‐1* sequences was performed as previously described.^[^ ^24^ ^]^ Briefly, ≈500 ng of genomic DNA was subjected to bisulfite mutagenesis using the EZ DNA Methylation‐Gold Kit (D5006, Zymo Research). A portion of bisulfite‐treated DNA samples was used for PCR amplification using specific primers designed as previously reported^[^ ^24^ ^]^ (Table S4, Supporting Information). The PCR products were then subjected to restriction digestion using restriction enzymes that recognize the specific CpG sites. HinfI (ER0801, Thermo Fisher) was used to digest bisulfite‐treated *LINE‐1*. After gel electrophoresis, the methylated and unmethylated DNA products displayed different patterns of restriction enzyme digestion.

### RNA‐seq and Relative Data Analysis

Total RNA was isolated from DAC‐treated THP‐1 cells using a total RNA Purification Kit (B518651, Sangon). RNA was assessed for quantity and quality using Agilent Bioanalyzer 2100 for a minimum RIN score of 7 or higher. cDNA libraries were prepared using RNA fragmentation, cDNA synthesis, ligation of index adaptors, and amplification using the KAPA mRNA HyperPrep Kit (KK8581, Roche). RNA‐seq was performed on the Illumina NovaSeq 6000 platform according to the manufacturer's instructions. Raw sequence reads were first processed using FastQC for quality control, then adapter sequences and poor‐quality reads were removed using Cutadapt (v1.9.1). Clean data were aligned to the reference human genome (hg38, downloaded from the Ensembl browser) using Hisat2 software (v2.1.0) and ordered using samtools (v1.6). HTSeq (v0.13.5) was then used to estimate gene and repeat element expression levels using different reference files. The gene reference file was GRCh38.gtf downloaded from the Ensembl browser and the repeat element reference file was hg38_rmsk_TE.gtf integrated by MHammell Lab.^[^ ^53^ ^]^ To discover differentially expressed dsRNAs, raw counts were analyzed using the R package DESeq2 (v1.28.1). The relative expression levels of genes in heatmaps are shown in Table S5 (Supporting Information). Gene Set Enrichment Analysis (GSEA) (v4.3.2) was used to analyze the enrichment of signaling pathways in different groups of samples. Volcano plots were generated using TBtools (v2.061).

### Bisulfite Sequencing PCR (BSP)

Genomic DNA was extracted as in COBRA and then sent to Tsingke Biotechnology Co., Ltd. for BSP. Six independent clones from each specimen were sequenced.

### Methylation‐Specific PCR (MSP)

Genomic DNA was extracted and then bisulfite modified as in COBRA. MSP primers were designed according to genomic sequences flanking the presumed transcription start sites for *RIG‐I*, *MDA5*, and *TLR3* (Table S4, Supporting Information). Primer sequences were oligo‐synthesized (IDT) to allow MSP to detect bisulfite‐induced changes affecting unmethylated (U) and methylated (M) alleles. Each MSP reaction incorporated ∼200 ng of bisulfite treated DNA as template, 400 nm of each primer, 2 × Taq Master Mix (Dye Plus) (P112‐01, Vazyme, China) in a final reaction volume of 10 µL. Cycle conditions were as follows: 95 °C, 5 min; 40 cycles, (95 °C, 20 s, 50 °C, 15 s, and 72 °C, 1 min); and 72 °C, 5 min. MSP products were analyzed with 1.5% agarose gel electrophoresis and stained with YeaRed dye (Yeasen, 10202ES76, China).

### Antibodies and Reagents

Antibodies used in this study are listed as follows: RIG‐I (3743S, Cell Signaling Technology), MAVS (sc‐166583, Santa Cruz Biotechnology), VDAC (55259‐1‐AP, Proteintech), IgG isotype control (3900S, Cell Signaling Technology), MX1 (A1780, ABclonal), RARα (A0370, ABclonal), CD45 (304007, BioLegend), GAPDH (AF0911, Affinity Biosciences), β‐actin (A00702, Genscript).

IFN‐α and IFN‐β were purchased from MedChemExpress. 5‐Azacytidine, AR7, library of FDA‐approved small‐molecule drugs and structural analogues to RARα agonists was purchased from Topscience. Fragments predicted to bind RARα were purchased from Specs.

### Immunoblotting

Immunoblotting was performed as previously reported.^[^ ^54^ ^]^ Briefly, cells were lysed in NP‐40 lysis buffer (1% SDS). Protein extracts were loaded on ExpressPlus PAGE gels (M42015C, Genscript). The gels were transferred to PVDF membranes, and the resulting blots were each incubated with a primary antibody and then with an appropriate HRP‐conjugated secondary antibody, followed by signal detection based on HRP activity.

### MAVS Aggregation Assays

MAVS aggregation was performed according to a published protocol.^[^ ^26^ ^]^ In brief, mitochondria were isolated using the Mitochondria isolation kit (89874, Thermo Fisher), and the mitochondria pellet was suspended in 1× sample buffer (0.5× TBE, 10% glycerol, 2% SDS and 0.0025% bromophenol blue) and subjected to semi‐denaturing detergent agarose gel electrophoresis (SDD‐AGE). Samples were loaded onto a 1.5% vertical agarose gel. After electrophoresis in running buffer (1× TBE and 0.1% SDS) for 40 min at a constant voltage of 80–100 V at 4 °C, proteins were transferred to PVDF membranes for immunoblotting.

### Real‐Time qRT‐PCR

Total RNA was isolated from cells using a Total RNA Purification Kit (B518651, Sangon), then 1 µg total RNA was reverse transcribed using HiScript II Q RT SuperMix for qPCR (+ gDNA wiper) (R223‐01, Vazyme) according to the manufacturer's protocol. PCR was performed in triplicate using ChamQ SYBR qPCR Master Mix (Low ROX Premixed) (Q331‐02/03, Vazyme) and a ViiA 7 Real‐Time PCR System (Applied Biosystems) under the following conditions: 5 min at 95 °C, followed by 40 cycles of 95 °C for 15 s and 60 °C for 60 s. Specificities of PCR products were verified for each primer set and sample by melting curve analysis. Gene expression levels were normalized relative to *ACTB* levels using the comparative Ct method. The primer sequences used are listed in Table S4 (Supporting Information).

### Retrovirus or Lentivirus Production and Infection

For RIG‐I retrovirus construction, 2 × 10^6^ HEK293T cells were seeded on a 10 cm dish for 16 h, followed by co‐transfection with two helper plasmids (6 µg pCAG‐VSVG and 9 µg pBS‐CMV‐gagpol) and 16 µg of the plasmid encoding RIG‐I constructed on a retroviral vector (MigR1) with IRES‐driven EGFP reporter. Transfection was performed using HilyMax transfection reagents (H357, Dojindo) according to the manufacturer's instructions, and the medium was replaced after 4 h. Retrovirus‐containing medium was collected 48 h post‐transfection. For the construction of RIG‐I‐overexpressing THP‐1 cells, 2 × 10^5^ THP‐1 cells were infected with RIG‐I retrovirus by spinning in a 6‐well plate at 2000 rpm for 2 h in the presence of 8 µg mL^−1^ polybrene (AL‐118, Sigma). After 6 h, an equal volume of fresh RPMI 1640 medium was added to the original virus‐containing medium. Seventy‐two hours post infection, infected cells (GFP‐positive cells) were purified by flow cytometry.

For RIG‐I shRNA lentivirus construction, 2 × 10^6^ HEK293T cells were seeded on a 10 cm dish for 16 h, followed by co‐transfection with two helper plasmids (6 µg pMD2.G and 9 µg psPAX2) and 16 µg of the plasmid encoding shRNA (shRIG‐I) constructed on a pLKO.1 puro lentivirus vector. Transfection was performed using HilyMax transfection reagents (H357, Dojindo) according to the manufacturer's instructions, and the medium was replaced after 4 h. Lentivirus‐containing medium was collected 48 h post‐transfection. For the construction of RIG‐I‐knockdown THP‐1 cells, 2 × 10^5^ THP‐1 cells were infected with RIG‐I shRNA lentivirus by spinning in a 6‐well plate at 2000 rpm for 2 h in the presence of 8 µg mL^−1^ polybrene (AL‐118, Sigma). After 6 h, an equal volume of fresh RPMI 1640 medium was added to the original virus‐containing medium. 72 h post‐infection, infected cells were selected with 2 µg mL^−1^ puromycin (T2219, Topscience) for 7‐10 days to establish stably expressing cell lines. The shRNA sequences are described in Table S4 (Supporting Information).

### Immunofluorescence Analysis

Cells were harvested and washed with PBS, then 1 × 10^5^ cells were centrifuged into the glass slides by cytospin at 800 rpm for 5 min. The cells were then fixed in 4% phosphate‐buffered saline (PBS)–paraformaldehyde for 15 min, incubated in 0.2% Triton X‐100 for 5 min, and then in 1% BSA in PBS for 10 min, and stained with RIG‐I or MX1 antibody for 1 h. Staining with a secondary antibody was performed for 20 min, followed by three washes. The cells were then shielded by Fluoroshield Mounting Medium with DAPI and visualized using an Olympus DP80 fluorescence microscope.

### Plasmid Construction and Transfection

Prof. Jiang Zhu provided mammalian expression plasmids for RIG‐I and RARα, which were verified by DNA sequencing. Lipofectamine 2000 (11668030, Thermo Fisher) was used to transfect the plasmids into cells.

### RNA Interference

Cells were transfected with siRNA using RFect siRNA transfection reagent (11012, Baidai) according to recommended procedures. The siRNA sequences are described in Table S4 (Supporting Information).

### Detection of Cytokine Production

The production and secretion of IFN‐α or IFN‐β in cell supernatants was measured using a human IFN‐α ELISA kit (BSEH‐084, Biosharp) and a human IFN‐β ELISA kit (BSEH‐274, Biosharp).

### Xenograft Assays

NCG (NOD‐*Prkdc* ^em26Cd52^ *Il2rg* ^em26Cd22^/NjuCrl) mice used in CDX studies were purchased from GemPharmatech Co. (Jiangsu, China) and housed under specific pathogen‐free conditions. Experiments were performed according to a Shanghai Jiao Tong University IACUC‐approved protocol.

For survival observation, DAC‐resistant THP‐1 cells (1 × 10^7^ cells) suspended in 100 µL PBS were injected intravenously into the tail vein of 6‐week‐old female NCG mice on day ‐7. Seven days (day 0) after tumor cells injection, mice were randomized into three groups (8 mice per group) and then intraperitoneally injected with PBS, 1 mg kg^−1^ DAC (T1508, Topscience) or 10 mg kg^−1^ ATRA (T1051, Topscience) daily. Mouse survival was monitored every 12 h and analyzed using the Log‐rank test (Mantel‐Cox).

To monitor disease progression, DAC‐resistant THP‐1 cells (1 × 10^7^ cells) suspended in 100 µL PBS were injected intravenously into the tail vein of 6‐week‐old female NCG mice on day ‐7. Seven days (day 0) after tumor cells injection, mice were randomized into three groups (3 mice per group) and then injected intraperitoneally with PBS, 1 mg kg^−1^ DAC or 10 mg kg^−1^ ATRA daily. These mice were sacrificed on day 17 and the spleens and PB were collected for detection.

### Flow Cytometric Analysis

Stained cells were analyzed on a BD LSRFortessa X‐20 flow cytometer (BD Biosciences). A monoclonal anti‐human CD45 antibody (304007, BioLegend) was used.

### Complete Blood Count Assay

PB samples were stored in EDTA tubes. Each sample was analyzed using an automated hematology instrument shortly after collection.

### Establishment of Potential RIG‐I Inducer Library

For 10,352 highly druggable fragments selected from the ZINC database (https://zinc.docking.org/), predicted docking affinities were calculated online using the mcule 1‐click Docking server (https://mcule.com/apps/1‐click‐docking/). For ∼760 000 small molecule compounds (∼180 000 natural products, ∼117 000 bioactive compounds, and ∼463 000 fragments) in the Topscience database (https://www.screeningcompound.com/), the structural similarity (Tanimoto index) to the eight established RARα agonists (TAM, AM580, tazarotene, adapalene, peretinoin, isotretinoin, ATRA and acitretin) was analyzed using R software. Details of the 102 collected potential RIG‐I‐inducing small molecules are shown in Table S2 (Supporting Information).

### Statistics

Statistical analysis (unpaired two‐tailed Student's t test, with 95% confidence interval under the untested assumption of normality; Fisher's exact test for categorical variables) was performed in GraphPad Prism 8.0. Data are presented as means ± SD. Group size was indicated in the main text. Significant differences between two groups were noted by asterisks (*P < 0.05; **P < 0.01; ***P < 0.001, NS, not significant).

## Conflict of Interest

The authors declare no conflict of interest.

## Author Contributions

X.‐Q.C., J.‐Q.W., Y.‐T.L., J.‐Y.H., and X.‐Q.W. contributed equally to this work. M.L. conceived the study. M.L. supervised the project. X.‐Q.C., J.‐Q.W., Y.‐T.L., J.‐Y.H., X.‐Q.W., J.‐L.W., S.‐J.X., H.‐X.S., Z.‐Y.W., N.Y., F.‐F.S., D.‐R.Z., K.T., H.‐S.Z., and J.‐Y.C. performed the experiments. S.‐J.Z., Wen Wu, Wei Wu, and J.‐Y.H. collected the primary samples. X.‐Q.C., Wei Wu, S.‐J.Z., and M.L. analyzed the data. X.‐Q.C., Wen Wu, S.‐J.Z., and M.L. wrote the manuscript.

## Supporting information



Supplemental Figures

Supplemental Tables

## Data Availability

RNA‐seq data have been deposited in the Gene Expression Omnibus database (GSE269185). The data that support the findings of this study are available from the corresponding author upon reasonable request.
