# Supplementary material for: RIG‐I‐Inducing Small Molecules Potently Inhibit HMA‐Resistant AML Through Igniting the Overloaded dsRNA Arsenal
Source: Adv Sci (Weinh). 2025 Jun 26;12(36):e14477. doi: 10.1002/advs.202414477 (PMC12463013; doi:10.1002/advs.202414477)

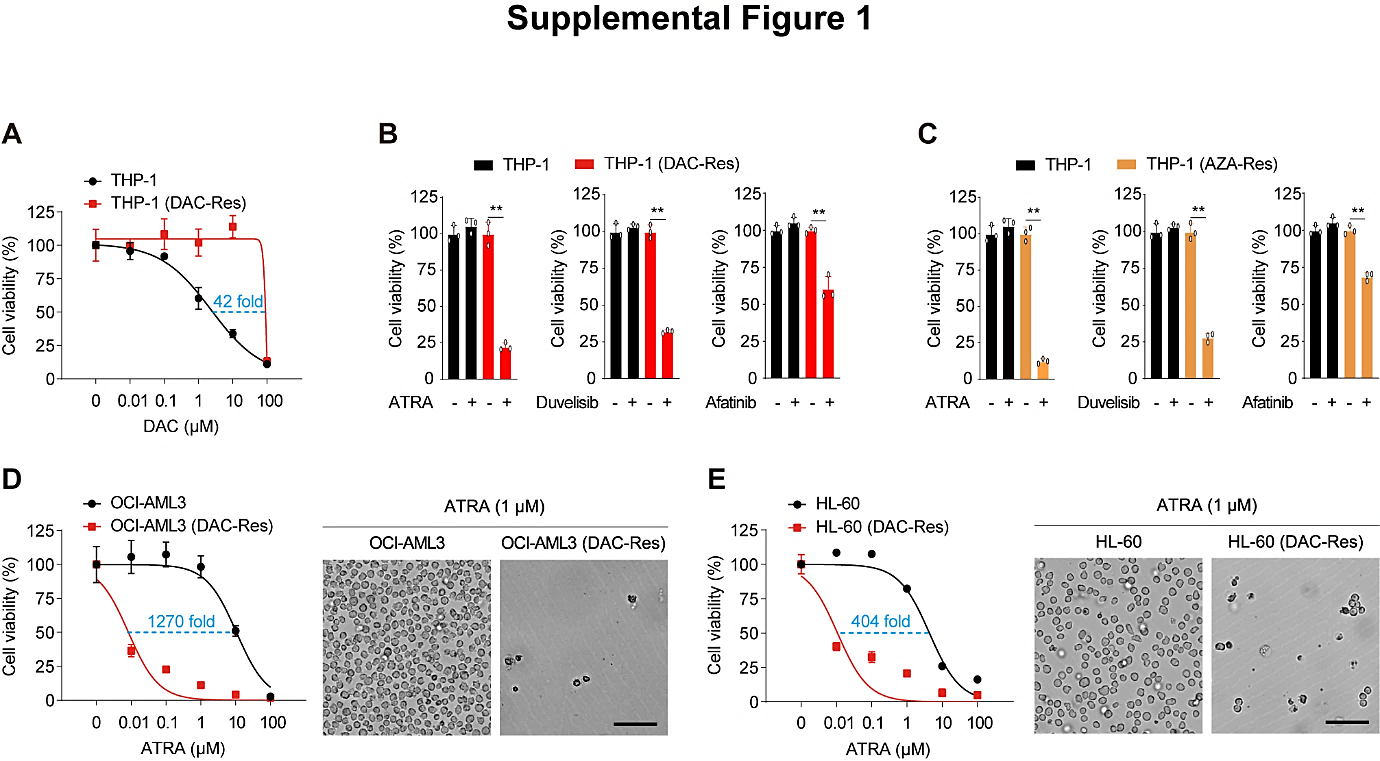


### Supplemental Figure 1. High-throughput screening identified ATRA as a potent and selective inhibitor of HMA-resistant AML cells.

(**A**) The indicated cells were treated with increasing doses of DAC for 72 hours, followed by determination of cell viability. (**B** and **C**) The parental, DAC-resistant (**B**) and AZA-resistant (**C**) cells were treated with 1 μM ATRA, duvelisib or afatinib for 72 hours, followed by cell viability assay. (**D**) Left panel, parental and DAC-resistant OCI-AML3 cell lines were treated with increasing doses of ATRA for 72 hours, followed by determination of cell viability. Right panel, phase-contrast images of the indicated cells treated with 1 μM ATRA for 72 hours. Scale bar, 50 μm. (**E**) Determinations on parental and DAC-resistant HL-60 cell lines as in (**D**). Error bars represent mean ± SD (n = 3 biological replicates, ***P* < 0.01).


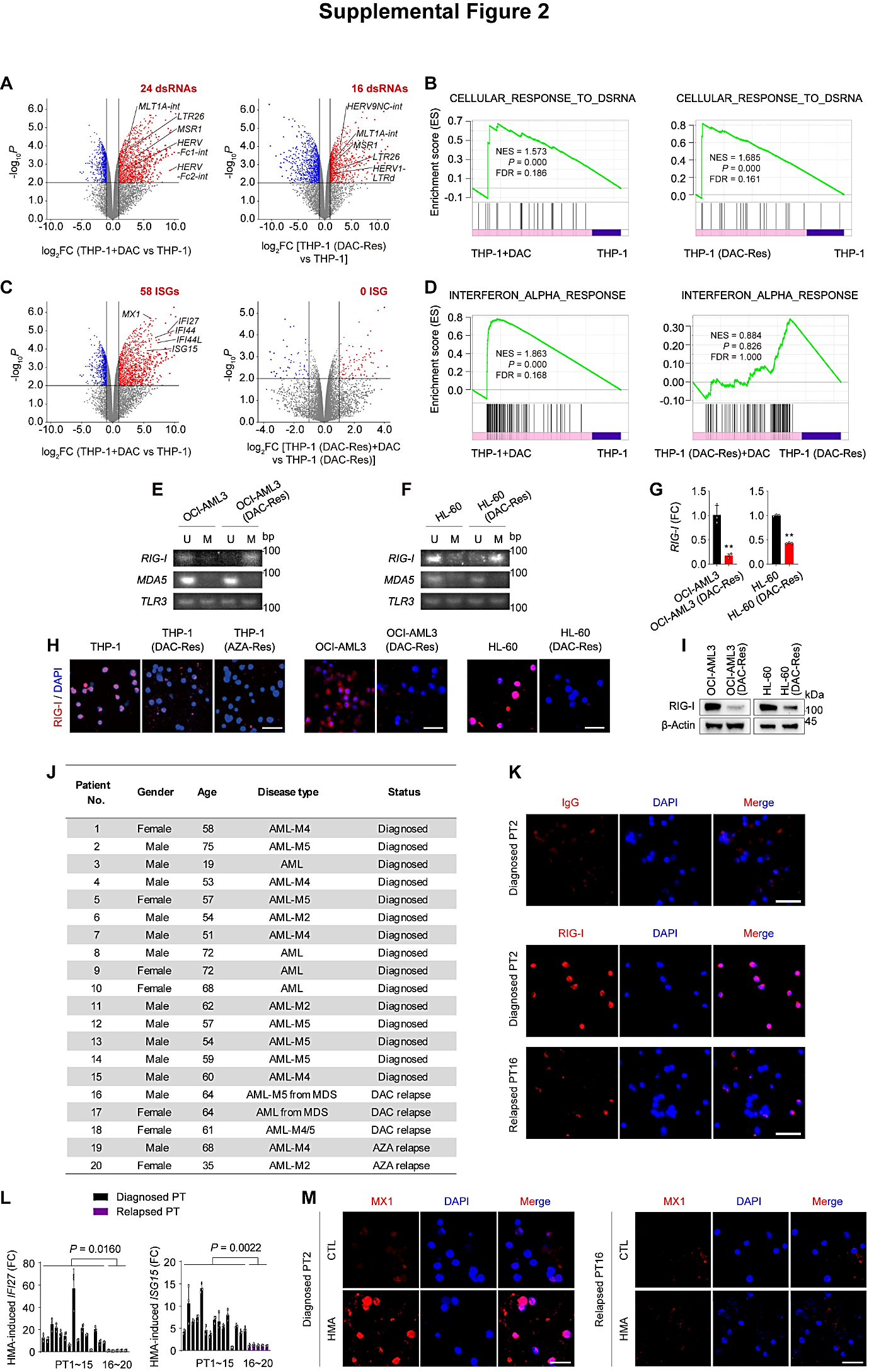


### Supplemental Figure 2. Downregulated RIG-I fails to trigger IFN anticancer immune response in HMA-resistant AML.

(**A**–**D**) RNA-seq data derived from **Figure 2C** were analyzed. (**A** and **C**) Volcano plot of differentially expressed genes (-log_10_*P* ≥ 2.0, log_2_FC ≥ 1.0 or ≤ -1.0) that are upregulated (red) or downregulated (blue) when comparing the indicated samples, including transcripts of dsRNAs (**A**) and ISGs (**C**). The representative and significantly upregulated dsRNAs and ISGs are labeled. (**B** and **D**) The enrichment score of the “cellular response to dsRNA” (**B**) and “interferon-alpha response” (**D**) signatures when comparing the indicated samples. The score was obtained by GSEA. NES, normalized enrichment score; FDR, false discovery rate. (**E** and **F**) Methylation-specific PCR detecting the indicated promoters in the indicated cells. U, unmethylated alleles; M, methylated alleles. (**G**) qRT-PCR determination of *RIG-I* mRNA levels in the indicated cells. (**H**) RIG-I immunostaining for the indicated cells. (**I**) Immunoblotting for RIG-I protein expression in the indicated cells. (**J**) Characteristics of the 20 patients followed for PBMC collection. (**K**) RIG-I immunostaining for the indicated primary PBMCs. (**L** and **M**) The indicated primary PBMCs were treated with 1 μM HMA for 72 hours. (**L**) qRT-PCR determination of *IFI27* and *ISG15*. (**M**) MX1 immunostaining of representative primary PBMCs. PT, patient. FC, fold change. Scale bar, 50 μm. Error bars represent mean ± SD (n = 3 biological replicates, ***P* < 0.01).


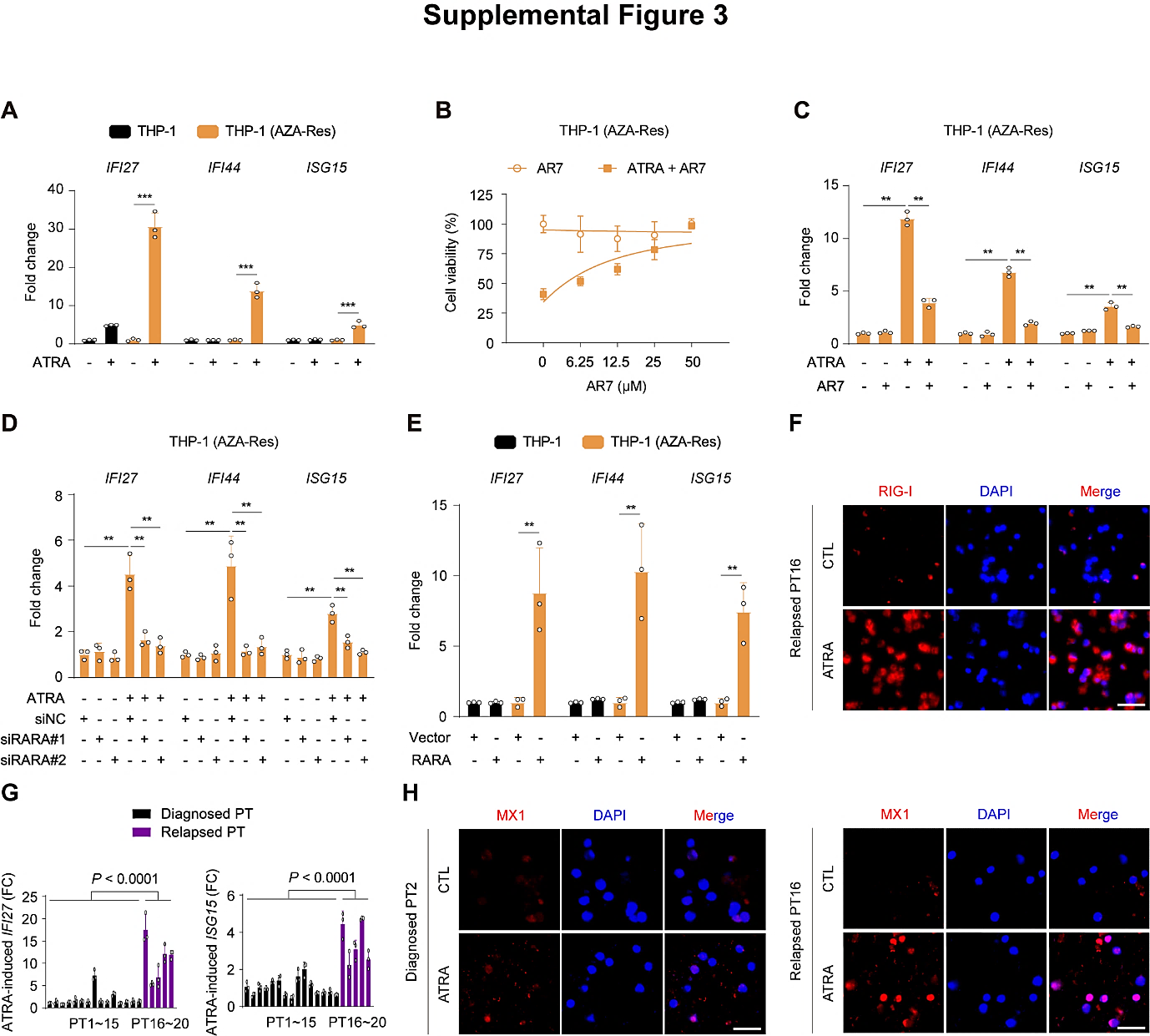


### Supplemental Figure 3. ATRA compensates for RIG-I expression and re-triggers IFN anticancer immune response in HMA-resistant AML.

(**A**) qRT-PCR determination of the mRNA levels of the indicated ISGs in the indicated cells after treatment with 1 µM ATRA for 72 hours. (**B**) The indicated cells were pretreated with increasing doses of AR7 for 4 hours, and then treated with or without 1 µM ATRA for 72 hours, followed by determination of cell viability. (**C**) The indicated cells were pretreated with 10 µM AR7 for 4 hours and then treated with 1 µM ATRA for 72 hours, followed by qRT-PCR determination of the mRNA levels of the indicated ISGs. (**D**) Cells were transfected with siRNA targeting RARα for 24 hours and then treated with 1 µM ATRA for 48 hours, followed by qRT-PCR determination. (**E**) Cells were transfected with empty vector or plasmid encoding RARα for 48 h, followed by qRT-PCR determination. (**F**–**H**) The indicated primary PBMCs were treated with 1 μM ATRA for 72 hours. (**F**) RIG-I immunostaining of the representative primary PBMCs. (**G**) qRT-PCR determination of *IFI27* and *ISG15*. (**H**) MX1 immunostaining of representative primary PBMCs. PT, patient. FC, fold change. Scale bar, 50 μm. Error bars represent mean ± SD (n = 3 biological replicates, ***P* < 0.01, ****P* < 0.001).


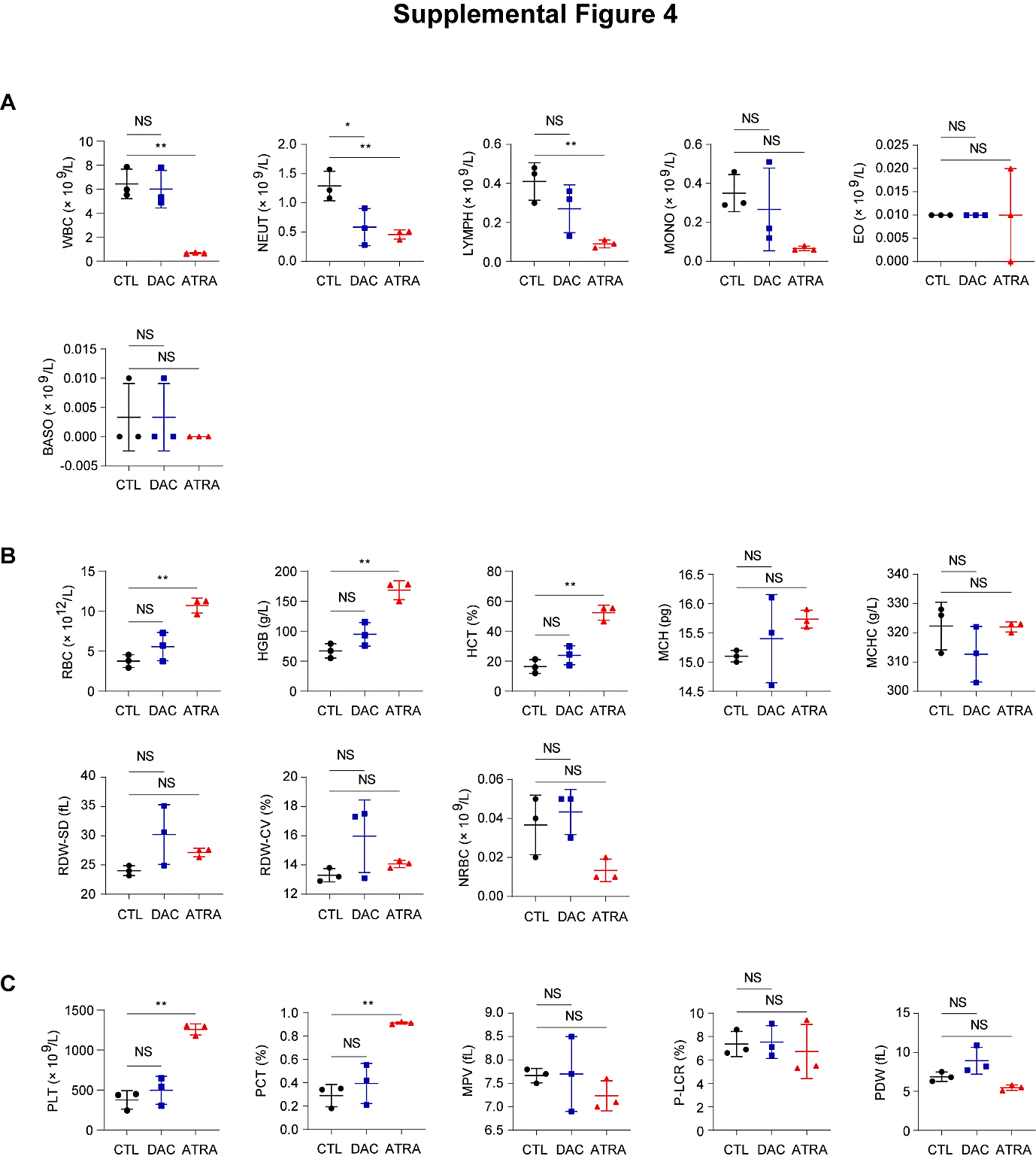


### Supplemental Figure 4. ATRA prolongs survival of HMA-resistant AML xenograft mice by re-triggering IFN anticancer immune response.

(**A**–**C**) The indicated indices of complete blood count for the PB in **Figure 4C**. (**A**) The indicated indices related to white blood cells (WBC). NEUT, neutrophils. LYMPH, lymphocytes. MONO, monocytes. EO, eosinophils. BASO, basophils. (**B**) The indicated indices related to red blood cells (RBC). HGB, hemoglobin. HCT, hematocrit. MCH, mean corpuscular hemoglobin. MCHC, mean corpuscular hemoglobin concentration. RDW-SD, red blood cell distribution width-standard deviation. RDW-CV, red blood cell distribution width-coefficient of variation. NRBC, nucleated red blood cells. (**C**) The indicated indices related to platelets (PLT). PCT, plateletcrit. MPV, mean platelet volume. P-LCR, platelet-large cell ratio. PDW, platelet distribution width. NS, not significant. Error bars represent mean ± SD (n = 3 biological replicates, **P* < 0.05, ***P* < 0.01).


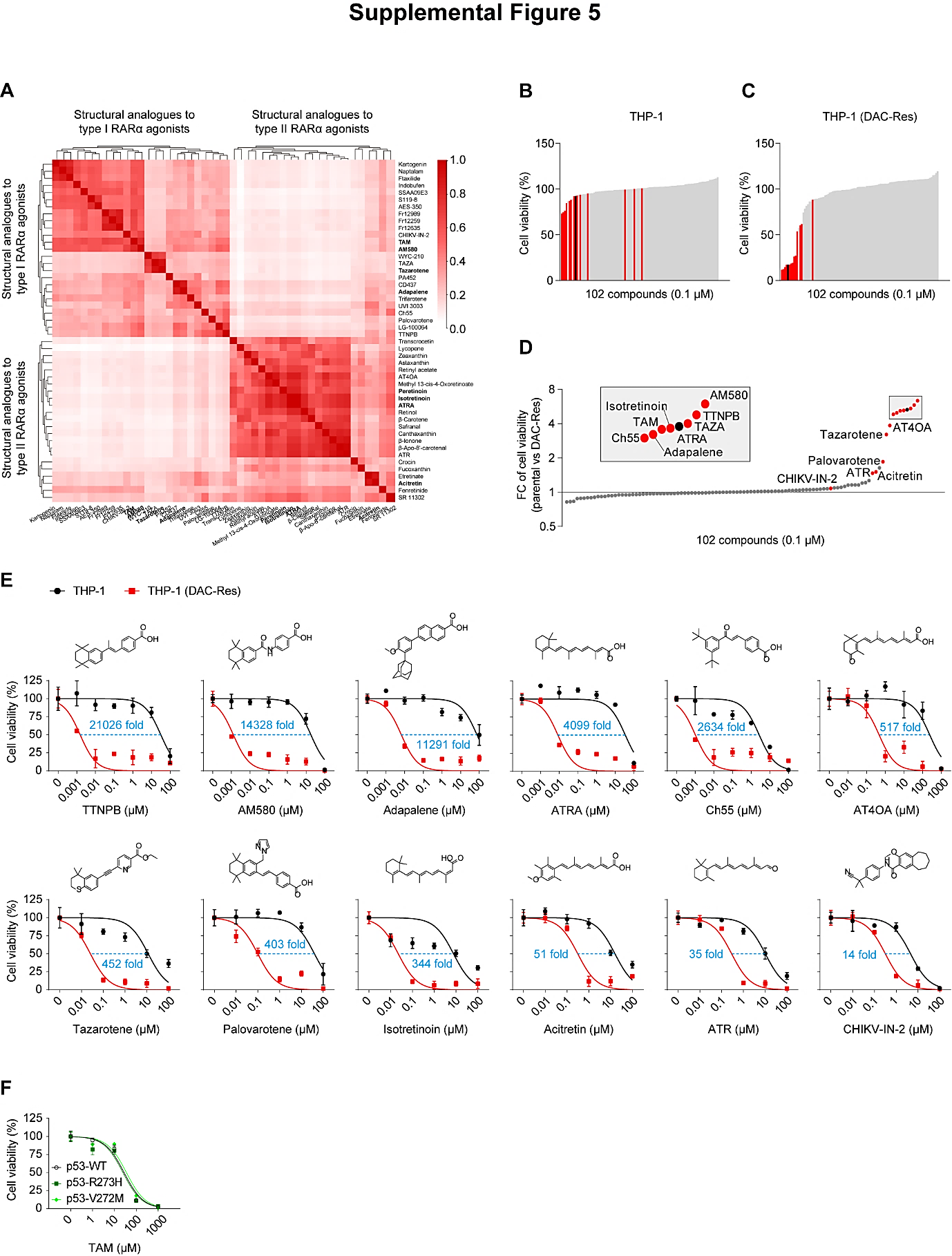


### Supplemental Figure 5. Rational identification of small molecules that kill HMA-resistant AML cells with super potency and selectivity.

(**A**) Tanimoto similarities of the collected 48 analogues of RARα agonists. The Tanimoto similarities of the given molecules in the form of SMILES were calculated using R software. (**B**–**D**) Results of the screening performed with 0.1 μM compound as shown in **Figure 5C**–**E**. The compounds exhibiting higher selectivity between the two cell lines at 1 μM concentration are shown as red bars or dots. (**E**) The indicated cells were treated with increasing doses of the indicated compounds for 72 hours and cell viability was determined. The IC_50_ shift (fold) was calculated. The chemical structure is shown above the curves. (**F**) The indicated SJSA-1 cells with different p53 status, generated via CRISPR genome-editing technology, were treated with increasing doses of TAM for 72 hours, followed by determination of cell viability. Error bars represent mean ± SD (n = 3 biological replicates).


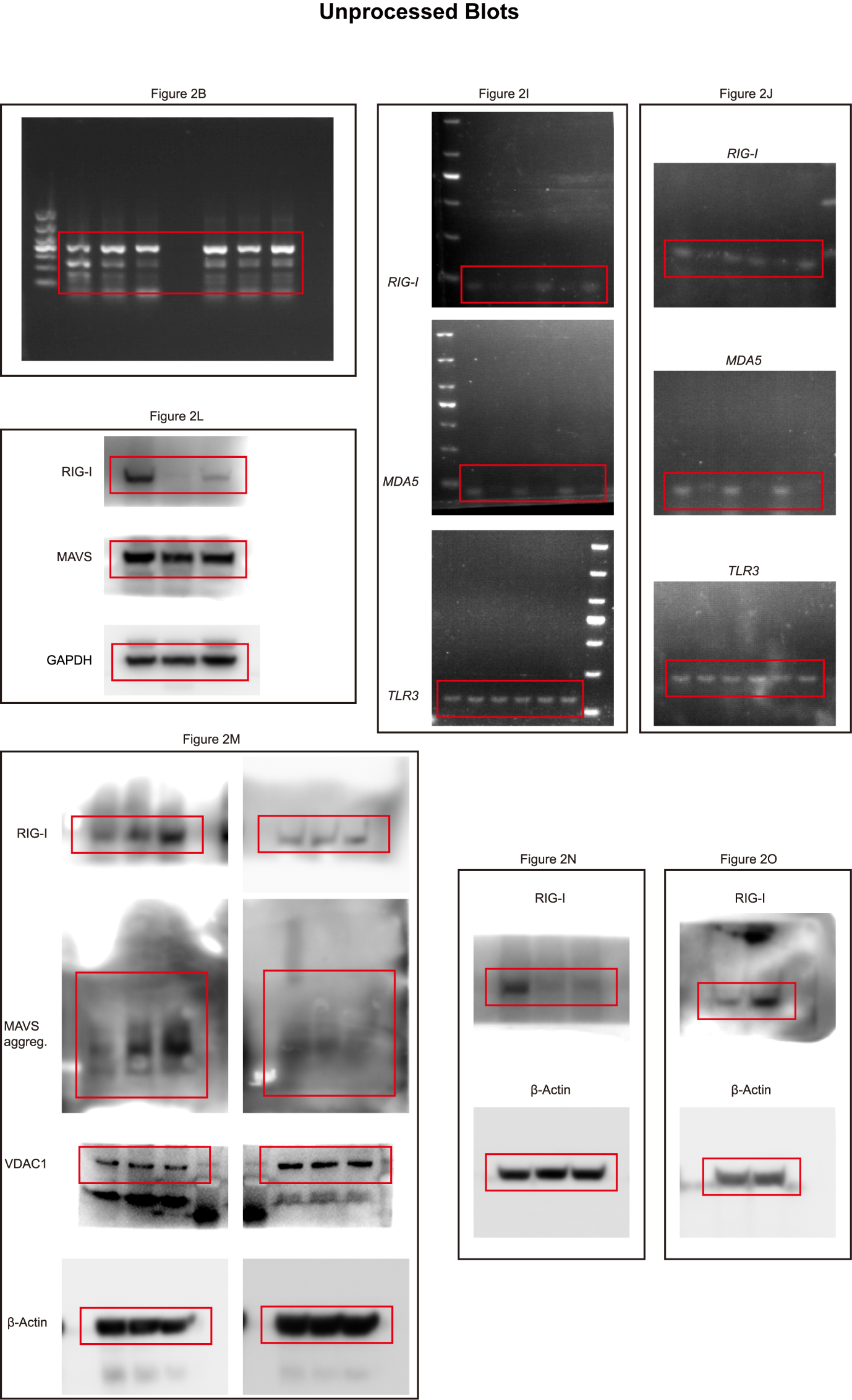


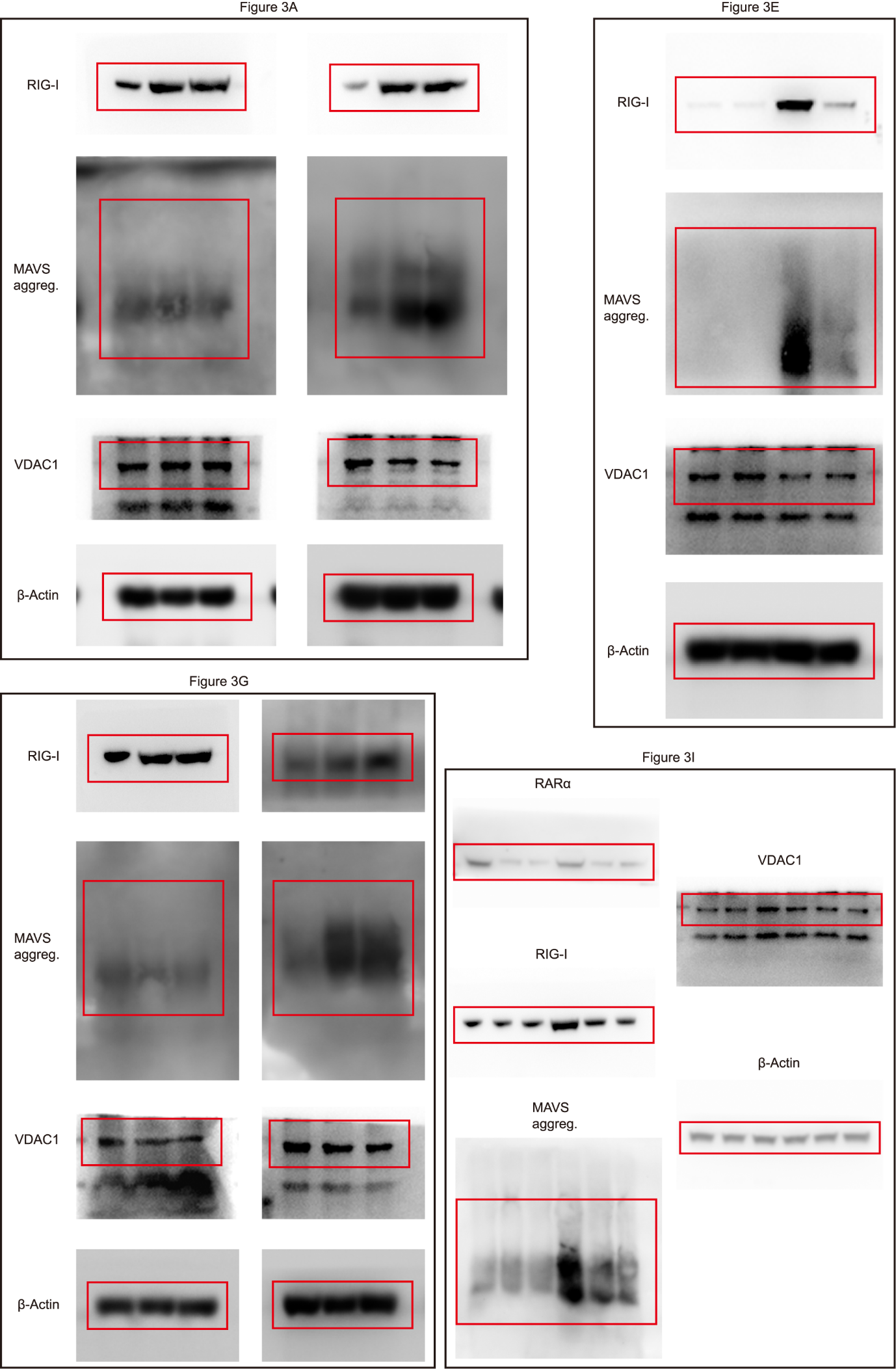


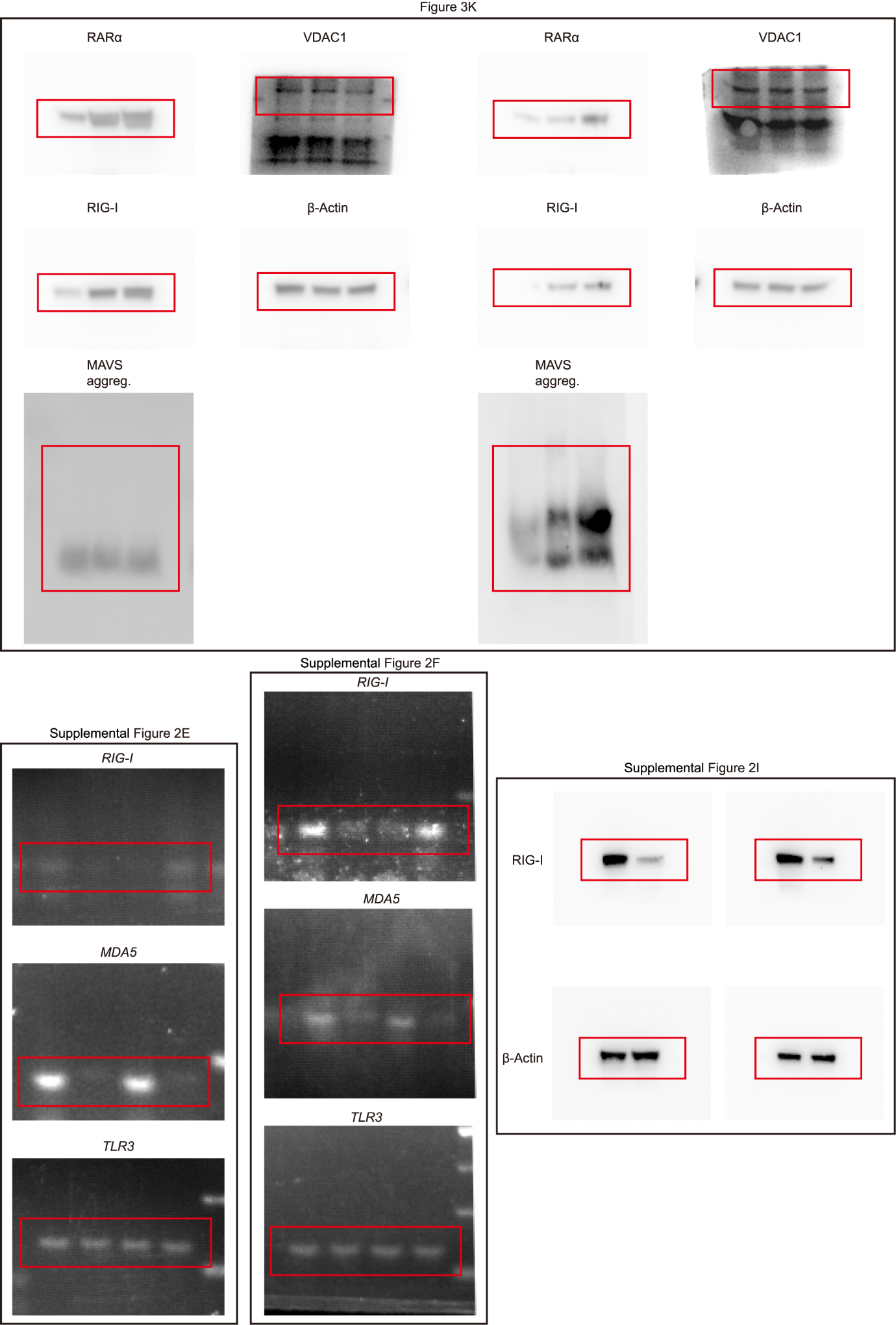

Supplement: Supplementary file 1 — Supplemental Figures [file ADVS-12-e14477-s002.docx]
